# Genome-wide association study of gastric cancer- and duodenal ulcer-derived *Helicobacter pylori* strains reveals discriminatory genetic variations and novel oncoprotein candidates

**DOI:** 10.1099/mgen.0.000680

**Published:** 2021-11-30

**Authors:** Vo Phuoc Tuan, Koji Yahara, Ho Dang Quy Dung, Tran Thanh Binh, Pham Huu Tung, Tran Dinh Tri, Ngo Phuong Minh Thuan, Vu Van Khien, Tran Thi Huyen Trang, Bui Hoang Phuc, Evariste Tshibangu-Kabamba, Takashi Matsumoto, Junko Akada, Rumiko Suzuki, Tadayoshi Okimoto, Masaaki Kodama, Kazunari Murakami, Hirokazu Yano, Masaki Fukuyo, Noriko Takahashi, Mototsugu Kato, Shin Nishiumi, Takashi Azuma, Yoshitoshi Ogura, Tetsuya Hayashi, Atsushi Toyoda, Ichizo Kobayashi, Yoshio Yamaoka

**Affiliations:** ^1^​ Department of Endoscopy, Cho Ray Hospital, Ho Chi Minh, Vietnam; ^2^​ Department of Environmental and Preventive Medicine, Oita University Faculty of Medicine, Oita, Japan; ^3^​ Antimicrobial Resistance ResearchCenter, National Institute of Infectious Diseases, Tokyo, Japan; ^4^​ Department of GI Endoscopy, 108 Central Hospital, Hanoi, Vietnam; ^5^​ Department of Molecular Biology, 108 Military Central Hospital, Hanoi, Vietnam; ^6^​ Department of Microbiology, Cho Ray Hospital, Ho Chi Minh, Vietnam; ^7^​ Department of Gastroenterology, Oita University Faculty of Medicine, Yufu, Oita, Japan; ^8^​ Department of Computational Biology and Medical Sciences, Graduate School of Frontier Sciences, University of Tokyo, Tokyo, Japan; ^9^​ Institute of Medical Science, University of Tokyo, Tokyo, Japan; ^10^​ Graduate School of Life Sciences, Tohoku University, Sendai, Japan; ^11^​ Department of Molecular Oncology, Chiba University, Chiba, Japan; ^12^​ Department of Infectious Diseases, Kyorin University School of Medicine, Mitaka City, Tokyo, Japan; ^13^​ Division of Endoscopy, Hokkaido University Hospital, Sapporo, Hokkaido, Japan; ^14^​ Department of Gastroenterology, National Hospital Organization Hakodate Hospital, Hakodate, Hokkaido, Japan; ^15^​ Department of Gastroenterology, Graduate School of Medicine, Kobe University, Chuou-ku, Kobe, Hyogo, Japan; ^16^​ Department of Omics Medicine, Hyogo College of Medicine, Hyogo, Japan; ^17^​ Department of Bacteriology, Faculty of Medical Sciences, Kyushu University, Fukuoka, Japan; ^18^​ Division of Microbiology, Department of Infectious Medicine, Kurume University School of Medicine, Kurume, Fukuoka, Japan; ^19^​ Advanced GenomicsCenter, National Institute of Genetics, Shizuoka, Japan; ^20^​ Research Center for Micro-Nano Technology, Hosei University, Tokyo, Japan; ^21^​ Department of Medicine, gastroenterology section, Baylor College of Medicine, Houston TX, USA

**Keywords:** duodenal ulcer, gastric cancer, GWAS, Helicobacter pylori, population genomics, recombination

## Abstract

Genome-wide association studies (GWASs) can reveal genetic variations associated with a phenotype in the absence of any hypothesis of candidate genes. The problem of false-positive sites linked with the responsible site might be bypassed in bacteria with a high homologous recombination rate, such as *Helicobacter pylori *, which causes gastric cancer. We conducted a small-sample GWAS (125 gastric cancer cases and 115 controls) followed by prediction of gastric cancer and control (duodenal ulcer) *H. pylori* strains. We identified 11 single nucleotide polymorphisms (eight amino acid changes) and three DNA motifs that, combined, allowed effective disease discrimination. They were often informative of the underlying molecular mechanisms, such as electric charge alteration at the ligand-binding pocket, alteration in subunit interaction, and mode-switching of DNA methylation. We also identified three novel virulence factors/oncoprotein candidates. These results provide both defined targets for further informatic and experimental analyses to gain insights into gastric cancer pathogenesis and a basis for identifying a set of biomarkers for distinguishing these *H. pylori *-related diseases.

## Data Summary

The authors confirm all supporting data, code, and protocols have been provided within the article or through supplementary data files. Assembled contigs of the 240 hspEAsia strains are available at https://figshare.com/s/2174da1fa20ae71c71e0. Sequencing and assembly statistics as well as other metadata of the newly sequenced strains are presented in Table S1, together with those of 405 other strains registered in public databases and initially analysed in this study. Data of the newly PacBio sequenced strains were deposited in DDBJ and mirrored in NCBI under BioProject accession number PRJDB5843. Data of newly HiSeq sequenced strains isolated in Vietnam were deposited in DDBJ and mirrored in NCBI under BioProject accession number PRJDB10671, and those isolated in Japan were also deposited under BioProject accession number PRJDB10720, PRJNA215152, PRJNA215153, PRJNA246665, and PRJNA246666 (Table S2). Raw output files of the SNP-GWAS and unitig-GWAS as well as a file of the phenotype of the strains (used as input to the GWAS programme *bugwas*) are available at https://figshare.com/articles/dataset/GWAS_of_gastric_cancer-_and_duodenal_ulcer-derived_H_pylori_strains_output_and_phenotype_files/15071838.

**Impact Statement**

Genome-wide association study aims to reveal a genetic variation associated with a phenotype through genome comparison; however, there is a problem of false positives due to linkage (correlation) with other genetic variations. An organism with a high rate of homologous recombination for breaking this linkage would be an ideal target to bypass this problem. We focused on such a bacterial species, *Helicobacter pylori,* a stomach pathogen infecting over half of the human population and causing gastritis and ulcers; constructed and analysed the dataset of hundreds of genomic sequences of *H. pylori* strains isolated from patients with gastric (stomach) cancer and duodenal ulcer; identified the genetic variations associated with phenotypic differences. Despite the small sample size and difficulty in applying the popular method based on unitigs (unique DNA substrings of variable length), we identified 11 single nucleotide polymorphisms (eight amino acid changes) and three DNA motifs that allowed effective discrimination and suggested the different molecular mechanisms underlying the differences. In addition to known virulence factors, we identified several novel candidates, each likely interfering with specific human cellular reactions. These results provide a basis for early-stage distinction between gastric cancer and duodenal ulcer, and for selecting an appropriate treatment, although its application in clinical settings requires further validation.

## Introduction

The faster-than-exponential decrease in the cost of DNA sequencing has brought about the era of population genomics, which refers to the comparative analysis of numerous genome sequences within a population. Among the various population genomics methods available, genome-wide association study (GWAS) has the advantage of being able to reveal genetic variations associated with a particular phenotype in the absence of any hypothesis of candidate genes. GWASs have revealed the genetic basis of various human diseases, including some with multiple genetic factors. Although GWAS in bacteria has been difficult due to the strong population structure [1], methodological developments in the last several years [1–3] have helped to control this effect and to systematically explore relationships between a phenotypic trait and any genetic variation, such as the presence/absence of specific genes or single nucleotide polymorphisms (SNPs). GWASs utilizing these methodological developments have identified the genetic basis of several bacterial activities, including host specificity [4], survival in different environments [5], and antimicrobial resistance [6, 7].

Berthenet *et al*. (2018) [8] conducted a GWAS on *Helicobacter pylori *, a stomach pathogen that infects more than half of the global population and causes gastric cancer. *H. pylori* has a very high recombination rate, leading to the generation of short recombination-derived chunks across the genome (median, 14 bp, interquartile range, 5–39 bp) [9]. Thus, we reasoned that this feature of *H. pylori* would make it an ideal subject for a GWAS because due to the high recombination, the responsible SNP would quickly be separated from linked SNPs that otherwise would appear as false positives (Fig. S1).

The population structure of *H. pylori* is distinctive because global strains are phylogeographically differentiated and classified into several populations (hpAfrica2, hpAfrica1, hpNEAfrica, hpEurope, hpAsia2, hpSahul, and hpEastAsia) that have notable genotypic and phenotypic differences [10]; for example, the frequency of strains carrying the *cag* pathogenicity island, the strongest risk factor for gastric cancer, is nearly 100 % in East Asia compared to ~60 % in other regions [11]. The population structure requires GWAS to be conducted separately for each population. The GWAS by Berthenet *et al*. (2018) [8] was conducted in the hpEurope population. On the other hand, hspEAsia, a subpopulation of hpEastAsia, is of special interest because it has been associated with the highest incidence of gastric cancer in East Asia [12]. This is often explained by the presence of East Asian-type CagA protein, which has distinctive sequence differences from Western CagA [13]. CagA is encoded in the *cag* pathogenicity island and is injected into host cells, where it interacts with a number of host proteins involved in cell signalling [14].

Molecular epidemiological studies have suggested that a single *H. pylori* virulence factor does not sufficiently explain its clinical outcomes [15]. In an attempt to explore other factors, Berthenet *et al*. (2018) [8] compared genome sequences of *H. pylori* strains isolated from patients diagnosed with non-atrophic gastritis (NAG), a step toward cancer, and gastric cancer patients, and reported that genes in the *cag* pathogenicity island and encoding an outer membrane protein BabA are typically associated with gastric cancer. However, the associations among genes in the *cag* pathogenicity island were as expected. In addition, the transition from NAG to gastric cancer is not discrete, but continuous, and the study did not elucidate how amino acid changes in the GWAS hits underlie the pathophysiological alterations.

Duodenal ulcer is also caused by *H. pylori *, but is considered divergent, and even somewhat mutually exclusive, from gastric cancer [16]. Duodenal ulcer arises from antral-predominant gastritis associated with excessive gastric acid secretion, whereas gastric cancer develops from a background of corpus-predominant gastritis or pangastritis, leading to hypochlorhydria as progressive atrophic gastritis occurs, and followed by intestinal metaplasia [17]. Cohort studies have revealed that patients with a history of duodenal ulcer have a considerably reduced risk of developing gastric cancer [18, 19]. A meta-analysis revealed that *dupA* is associated with an increased risk of duodenal ulcer, but a decreased risk of gastric cancer [20]. A study of duodenal ulcer and gastric cancer in a Moroccan population identified some specific genotypes of the virulence genes *cagA* and *vacA*s to be strongly associated with the risk of gastric cancer or duodenal ulcer development [21]. However, another study failed to detect any association between genetic factors and phenotypic traits of gastric cancer/duodenal ulcer [22]. These previous studies, focusing on specific genes, did not account for the population structure of *H. pylori* strains, which would have inevitably resulted in overestimation of the extent of association [1]. Moreover, no genome-wide study has explored a genetic marker for the discrimination of gastric cancer and duodenal ulcer. The discrimination at an early-stage is important because the treatment guideline is different between gastric cancer and duodenal ulcer: duodenal ulcer can be completely cured by eradicating *H. pylori* from the host, whereas gastric cancer requires treatment of a tumour (e.g., resection) in addition to eradicating *H. pylori *, and annual follow-up using endoscopy.

In this study, we focused on duodenal ulcer as a reference for comparison with gastric cancer to answer the key question, what are the (potentially multiple) underlying genotypic factors in *H. pylori* that determine the risk of gastric cancer versus duodenal ulcer development in infected patients and would allow for differentiating between them? Unravelling these factors would be vital in understanding the microevolutionary processes toward duodenal ulcer/gastric cancer pathogenesis and would aid the development of clinical and epidemiological applications. To this end, we conducted a GWAS followed by prediction of a large number of *H. pylori* strains isolated from gastric cancer and duodenal ulcer patients in an hspEAsia subpopulation, revealing key discriminatory genetic variations and novel oncoprotein candidates.

## Methods

### Bacterial isolation and genome sequencing and assembly

*H. pylori* strains were isolated in Vietnam (from patients indicated for upper endoscopy at Cho Ray Hospital, Ho-Chi Minh and 108 Military Hospital, Hanoi) and in Oita, Japan, using standard culture methods. Briefly, homogenized antral biopsy specimens were inoculated on *H. pylori *-selective plates (Nissui Pharmaceutical Co., Ltd.) and incubated at 37 °C in a microaerophilic condition for 3–10 days. Purple colonies that appeared were subcultured in Brucella Broth (Becton, Dickinson and Company) supplemented with 7 % horse blood. DNA was extracted using a DNeasy Blood and Tissue Kit (Qiagen Inc.). DNA concentrations were measured using a Quantus Fluorometer (Promega). High-throughput genome sequencing was performed on a HiSeq 2500 (2×100 or 2×150 paired-end reads) or MiSeq (2×300 paired-end reads) sequencer (Illumina), following the manufacturer’s instructions. Trimmomatic v0.35 was used to remove adapter sequences and low-quality bases from the raw short-read data [23]. Trimmed reads were *de novo* assembled to produce contigs using the SPAdes (v3.12.0) genome assembler with the ‘-careful’ option to reduce mismatches in the assembly [24]. The minimum contig length was set to 200 bp.

Glycerol stocks of *H. pylori* strains isolated in Japan (from patients in different geographical areas of Japan, including Fukui, Hokkaido, Okinawa, and Oita) were propagated on trypticase soy agar supplemented with 5 % sheep blood (BD Biosciences) at 37 °C under microaerobic (5 % O_2_) conditions in a HERAcell 150i CO_2_ incubator (Thermo Fisher Scientific). *H. pylori* colonies were pooled, transferred into a Petri dish containing 40 ml of Brucella Broth supplemented with 10 % foetal bovine serum (Sigma-Aldrich), and incubated under agitation for 3 days. After incubation, the cells were harvested in 50 ml tubes and frozen. Genomic DNA was extracted from the frozen cell pellets using Qiagen Genomic-tip 100 G^−1^, RNase A, Proteinase K, and Genomic DNA Buffer Set (all from Qiagen), essentially following the protocol described in the Qiagen Genomic DNA Handbook. Genomic DNA was resuspended in TE buffer and sheared for library construction using a Covaris g-TUBE device according to the manufacturer’s instructions. A SMRTbell library was prepared using a SMRTbell Template Prep Kit 1.0 (Pacific Biosciences). DNA fragments larger than 17 kbp were size-selected using the BluePippin system (Sage Science). For each *H. pylori* strain, one SMRT cell was run on the PacBio RS II System with P6/C4 or P6/C4v2 chemistry and 360 min movies (Pacific Biosciences). SMRT sequencing data were analysed using SMRT Analysis v2.3.0 through the SMRT Portal. Reads were assembled using RS_HGAP_Assembly.2. After the removal of overlapping ends, the chromosomal contig was reshaped to start from the ori-sequence. Thereafter, it was re-sequenced with RS_Resequencing.1 to create consensus sequences.

### Population assignment of each strain

We inferred the population structure of 614 global strains in total, using chromosome painting and fineSTRUCTURE, as previously described [25]. Briefly, a contig from each genome was initially mapped to the genome of strain 26 695 as a reference, using Snippy v4.0.7 (https://github.com/tseemann/snippy). The Snippy-core function was used to create genome-wide haplotype data for all strains. Subsequently, ChromoPainter (v0.04) inferred chunks of DNA donated from a donor to a recipient for each recipient haplotype and summarized the results into a co-ancestry matrix. Using this co-ancestry matrix, fineSTRUCTURE (v0.02) then clustered individuals by a Bayesian Markov chain Monte Carlo (MCMC) approach with 100 000 iterations for both the burn-in and the MCMC chain after the burn-in.

### GWAS

All isolates assigned to hspEAsia based on the fineSTRUCTURE results and for which clinical information of interest (gastric cancer or duodenal ulcer) was available were used for GWAS. First, a maximum-likelihood phylogenetic tree based on core-genome SNPs was reconstructed using PhyML [26], and the distribution of gastric cancer and duodenal ulcer in the tree was visualized using Phandango [27]. The tree is shown as mid-point-rooted. Core-genome SNPs were extracted based on mapping of each genome against that of the East Asian-type (hspEAsia) *H. pylori* strain F57, using Snippy v4.0.7. We used strain F57 as a reference because it was isolated from a gastric cancer patient in Japan and its genome sequence has been determined by whole-genome shotgun sequencing [28].

Next, we conducted a pairwise genome alignment between each genome and strain F57 using progressiveMauve [29], which enables the construction of positional homology alignments even for genomes with variable gene content and rearrangement. Subsequently, we combined all alignments into a multiple genome alignment in which each position corresponded to that of the strain F57 reference genome. Next, we extracted SNPs with ≤10 % missing frequency and >5 % minor allele frequency. We conducted a SNP-GWAS based on a previous study [8], in which a linear mixed regression model with the *bugwas* package [30] was used to control for population structure based on an *n*×n relatedness matrix calculated from SNPs. We also conducted a SNP-GWAS in which the algorithm-factored spectrally transformed linear mixed model (FaST-LMM) implemented in pyseer [3] was used to control for population structure from the same set of SNPs. A Q-Q plot was created using the R statistical programme to assess the number and magnitude of observed associations between SNPs and disease (gastric cancer and duodenal ulcer) as compared to the association statistics expected under the null hypothesis of no association. We then conducted a permutation test in which in each of 100 permutations, the phenotype was randomly reassigned to each strain while maintaining the true genotype, and the average number of SNPs showing *P*-values less than a threshold by chance was counted, so as to exclude the same number of candidates from the top-hits in the Q-Q plot for further analysis.

We also conducted a GWAS in which unitigs were counted using unitig-counter [2], and unitigs with >5 % minor allele frequency were tested using the *bugwas* package and pyseer. A Q-Q plot was created, and a permutation test was conducted in the same way as SNP-GWAS. For top-hit unitigs, we conducted mapping to the reference genome, grouping, and annotation according to a recent study on *N. gonorrhoeae* [31].

In both the SNP-GWAS and unitig-GWAS, the heritability score (h^2^) was calculated for each SNP or unitig in pyseer. Conducting unitig-GWAS as well as SNP-GWAS would not particularly affect the multiple testing threshold because essentially the same correlated genetic changes are being tested.

In addition, we conducted a GWAS focusing on the presence or absence of specific genes rather than SNPs, based on pan-genome analysis using the panaroo pipeline with ‘--clean-mode sensitive -c 0.9’ option [32]. A matrix of presence or absence of genes with >5 % minor allele frequency was used as an input of the linear mixed regression model implemented in the *bugwas* package.

### Discrimination between gastric cancer and duodenal ulcer using a set of SNPs and DNA motifs identified by GWAS

Top-hit SNPs and DNA motifs deviating from the null hypothesis in the Q-Q plot and associated with gastric cancer were used to calculate a simple discriminatory score for each strain [8]. For each SNP and DNA motif, (*j*=1,…, *N*), where *N* indicates the number of top-hit SNPs and DNA motifs, when it has a nucleotide or DNA motif that increases in frequency in gastric cancer, a variable *I_j_* is set to 1 if the strain has it or –1 if it does not.

We used a random forest model to predict the probability of a strain *i* (*p_i_*) being isolated from a gastric cancer patient using a set of $I_{j}\times -{log}_{10}\left(P_{j}\right)$ as explanatory variables (where $P_{j}$ is *P*-value of the *j*-th SNP or DNA motif).

We then conducted a two-fold cross-validation in which the random forest model was fit to a training dataset, and the probability for each strain in a test dataset ( ${\hat{p}}_{i}$ ) was predicted from the explanatory variables of a strain *i*. Receiver operating characteristic (ROC) curves were drawn from the true host disease status (gastric cancer or duodenal ulcer) and the predicted probability ( ${\hat{p}}_{i}$ ) of each strain to calculate the area under the ROC curve (AUC), determine the optimal cutoff value of ${\hat{p}}_{i}$ to obtain the point closest to the top-left part of the ROC plot for the discrimination of gastric cancer or duodenal ulcer, and calculate the false positive and false negative rate of the discrimination under the optimal cutoff, using the R package pROC [33]. Training and test datasets were prepared (see Results) to check the robustness of results.

### Analysis of amino acid and RNA changes at SNPs

Non-synonymous SNPs deviating from the null hypothesis were mapped on 3D structural models of their protein products (in strain F57 or 26695), using the automated homology modelling programmes SWISS-MODEL (https://swissmodel.expasy.org/interactive) and PyMOL (Molecular Graphics System, v.1.2r3pre, Schrödinger, LLC). We also used KEGG (https://www.genome.jp/kegg/), UniProt (https://www.uniprot.org/), RCSB (https://www.rcsb.org/), SignalP-5.0 (http://www.cbs.dtu.dk/services/SignalP/), and cNLS Mapper (http://nls-mapper.iab.keio.ac.jp).

For intergenic SNPs deviating from the null hypothesis, we examined whether they were located in small regulatory RNAs previously identified in the reference strain 26695 [34] and registered in BSRD database [35] (http://kwanlab.bio.cuhk.edu.hk/BSRD/). M-fold (http://unafold.rna.albany.edu/?q=mfold) was used for secondary structure prediction.

## Results

### *H. pylori* strains from gastric cancer and duodenal ulcer in East Asia, and their population structure

**Fig. 1.**
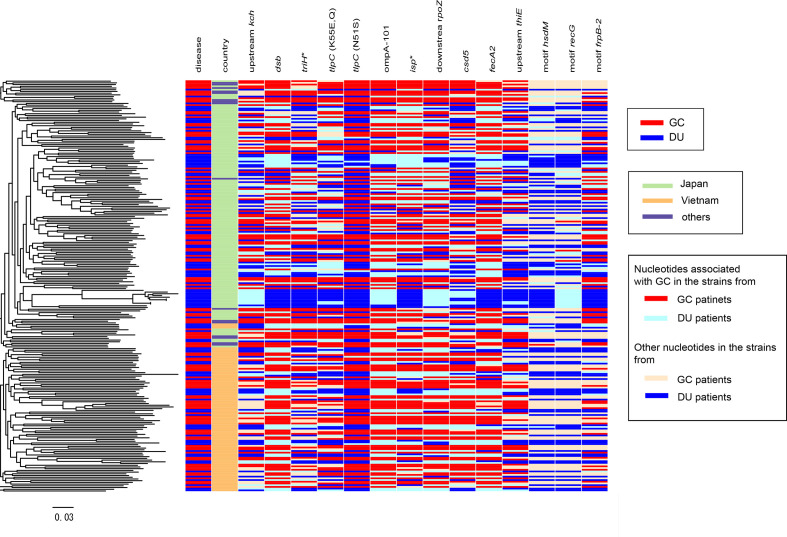
Core-genome phylogeny and metadata of the 240 strains from an hspEAsia population. Left: mid-point rooted core-genome phylogeny. Heatmap: column 1, host disease status (DU or GC). Column 2: country of isolation. Columns 3–14 correspond to the genes carrying a nucleotide associated with GC. GC, gastric cancer; DU, duodenal ulcer.

We combined genome data of 209 newly sequenced strains isolated in Vietnam and Japan with those of 383 strains representing the diverse global subpopulations [36] and those of 22 additional strains registered in the National Centre for Biotechnology Information (NCBI) GenBank repository with information on host disease status (gastric cancer or duodenal ulcer) (Table S1). We used the ChromoPainter/fineSTRUCTURE pipeline [25] to identify 11 clusters that were found in a previous study [36] (Fig. S2). After removing strains that were either unassigned to hspEAsia or without information of host disease status, 240 hspEAsia strains (125 gastric cancer and 115 duodenal ulcer) were selected. Among them, 137 isolates were from Japan, 87 from Vietnam, eight from Singapore, five from China, one from South Korea, one from Malaysia, and one from an unknown source (Table S2). A maximum-likelihood tree constructed based on core-genome SNPs revealed that, despite the substantial population structure, no cluster was solely associated with one disease status (gastric cancer or duodenal ulcer) (Fig. 1). The tree comprised two large clusters of mostly Japanese and Vietnamese (light green and orange in the second column ‘country’ in Fig. 1, respectively) strains that corresponded to two hspEAsia subpopulations in the fineSTRUCTURE results (Fig. S2), although there was no significant difference in disease status frequency between these clusters (*P*=0.2, Chi-square test). The large clusters of mostly Japanese and Vietnamese strains are reliable because the branch connecting them has >90 % bootstrap value (Fig. S3).

### GWAS identifies 11 SNPs and three DNA motifs associated with gastric cancer

**Fig. 2.**
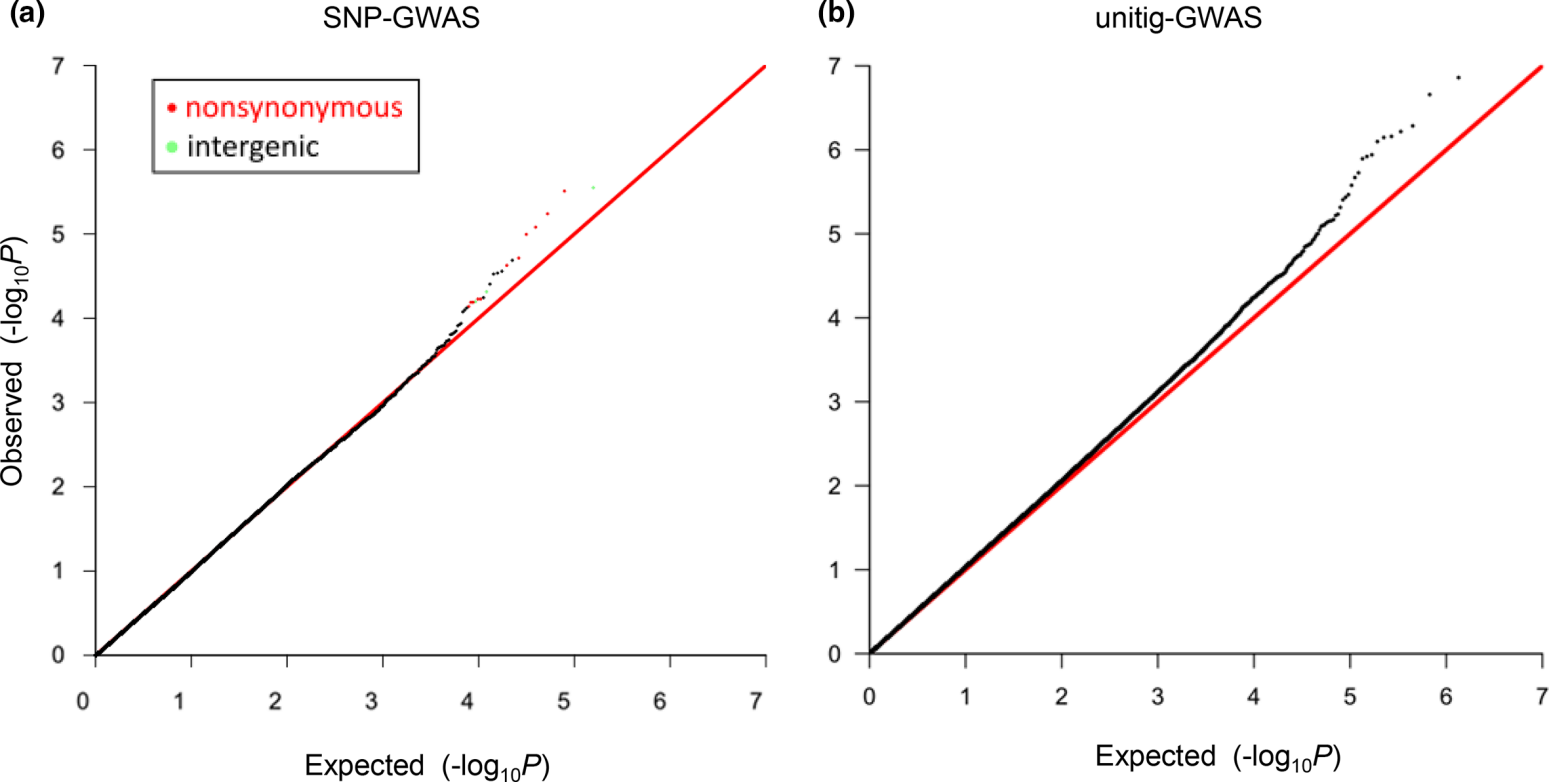
Q-Q plot to assess the GWAS results. Each dot in (a) indicates a SNP, whereas that in (b) indicates a unitig. Y-axis: observed $-\log_{10}(P)$ of each SNP or unitig, where *P* is its *P*-value. X-axis: expected $-\log_{10}(P)$ under the null hypothesis of no association. Non-synonymous and intergenic SNPs with *P*<10^-4^ and associated with GC are presented as red and green, respectively. GC, gastric cancer; SNP, single nucleotide polymorphism.

After adjustment for population structure and targeting 157 447 SNPs with a minor allele frequency >5 %, the SNP-GWAS using the *bugwas* package showed that the *P*-values of most SNPs were as expected under the null hypothesis of no association (Q-Q plot in Fig. 2a). This indicates the absence of systematic inflation of *P*-values after adjustment for population structure (i.e., genetic relatedness among strains in hspEAsia). At the same time, we found 23 outlier SNPs with *P*<10^−4^ deviating from the null hypothesis (Tables 1 and 2, Fig. 2a). The permutation test to confirm significance of the outlier SNPs showed that the number of SNPs showing *P*<10^−4^ under the randomly permuted phenotypes was on average 12. We thus excluded 12 SNPs (all nine synonymous SNPs and three less significant non-synonymous SNPs with lower -log10 (*P*) (Table 2) from the 23 SNPs and used remaining 11 SNPs (Table 1) for further analyses.

**Table 1.** Genetic variations associated with gastric cancer identified by GWAS.

Type	-log10(P)	Genomic position	Locus including or closest to the SNP	Gene name	Description	Function	Variant associated with gastric cancer	Position in the gene	Amino acid change	Corresponding locus in the strain 26 695
SNP	5.5	533 482	91 bp upstream of HPF57_0521 [532 255–533391] (-)	*kch, trkA*	potassium channel	potassium conductance regulator	G			HP0490
SNP	5.5	256 111	HPF57_0250 [255 679–256476] (+)	*dsbG/K*	thiol:disulfide interchange protein	disulfide bond formation for secretion	A	433	K145E	HP0231
SNP	5.2	155 782	HPF57_0151 [155 438–156307] (+)	*triH*	BIR, Dps/NapA, RAD21 similarity	host interference?	A	345	K115K,X	HP0130
SNP	5.1	96 796	HPF57_0094 [95 423–96958] (-)	*tlpC*	chemotaxis sensor	chemotaxis	A	163	K55E,Q	HP0082
SNP	5.0	1 459 449	HPF57_1382 [1 459 397–1460092] (+)	*ompA101*	outer membrane protein of OmpA family	uptake and outer membrane structure	T	53	V53A	HP1467
SNP	4.7	96 807	HPF57_0094 [95 423–96958] (-)	*tlpC*	methyl-accepting chemotaxis sensor	chemotaxis	C	152	N51S	HP0082
SNP	4.6	1 434 839	HPF57_1355 [1 434 577–1435371] (-)	*isp*	inactive Ser protease	inhibitor of proteases/chaperones?	G	533	G178E	HP1440
SNP	4.3	904 207	14 bp downstream of HPF57_0865	*thiE*	thiamine-phosphate synthase	supplying vitamin B1	A			HP0776
SNP	4.2	1 296 088	HPF57_1209 [1 295 855–1296457] (-)	*csd5*	cell shape determinant	helical cell shape	A	370	N124H,Y,D	HP1250
SNP	4.2	871 135	HPF57_0829 [870 769–873144] (-)	*fecA-2*	iron importer in outer membrane	iron uptake	C	2010	S670X,S	HP0807
SNP	4.2	839 132	29 bp upstream of HPF57_0798 [838 879–839103] (-)	*rpoZ*	RNA polymerase subunit omega	prophage silencing? stringenet control?	A			HP0915
motif	6.9	498631–498661	HPF57_0490 [497 344–498975] (-)	*hsdM*	Type I restriction enzyme M protein	DNA methyltransferase	not TAACGATAAC GATTTACACCT AAAGCTAGAC	315–345	multiple e.g. D115D,X	HP0463
motif	5.6	1522425–1522455	HPF57_1436 [1 522 369–1524234] (-)	*recG*	DNA recombinase		not ATTGACTTAG CCAAAGATGA AAACATTATCG	1780–1810	multiple	HP1523
motif	5.5	978037–978077	HPF57_0925 [978 055–980244] (-)	*frpB-2*	iron importer in outer membrane		TTGAAATTTC TTATAAGTTT TAATAATGGA TCTAAAAATGA	2168- C terminus-18bp downstream	multiple	HP_0915

position in F57 reference genome.

∗designated in this work.

isp, inactive serine protease; triH, triple halves.

**Table 2.** Synonymous SNPs and less significant non-synonymous SNPs identified by GWAS.

-log10(P)	Genomic position	Locus including or closest to the SNP	Gene name	Description	Function	Variant associated with gastric cancer	Position in the gene	Amino acid change	Corresponding locus in the strain 26695
4.7	485 026	HPF57_0479 [483 859–485079] (-)	*ispDF*	bifunctional 2-C-methyl-d-erythritol 4-phosphate cytidylyltransferase/2-C-methyl-d-erythritol 2,4-cyclodiphosphate synthase protein for isoprenoid synthesis	isoprenoid synthesis	T	54	–	HP1020
4.6	549 551	HPF57_0538 [549 128–550432] (+)	*pgbA*	plasminogen binding protein	anti-immunity	C	424	–	HP0508
4.5	163 266	HPF57_0158 [162 843–164288] (-)	*lldF*	L-lactate dehydrogenase to pyruvate	anaerobic catabolism	C	1023	–	HP0138
4.5	713 996	HPF57_0678 [711 939–714599] (+)	*bamA*	outer membrane protein assembly factor; surface antigen D15	assembly of outer membrane β-barrel proteins	C	2058	–	HP0655
4.4	101 839	HPF57_0101 [101 587–103584] (-)	*rpoD*	RNA polymerase sigma factor σ70	transcription initiation	C	1746	–	HP0088
4.2	389 519	HPF57_0382 [389 375–390157] (-)	*nadE*	NH_3_-dependent NAD^+^ synthetase	NAD synthesis	G	639	–	HP0329
4.2	598 098	HPF57_0574 [594 999–598511] (+)	*cagA*	cytotoxin-associated gene A	interferences with signal transduction	G	3100	A1034T,X,S	HP0547
4.2	146 365	HPF57_0139 [145 564–146508] (-)	*ctbP*	human C-terminal binding protein homolog	interference with gene expression? oncoprotein?	G	144	E48EDX	HP0096
4.1	1 102 964	HPF57_1035 [1 102 345– 1 103 568] (+)	*zmp*	zinc-metallo protease acting on isoprenylated protein		A	620	K207R	HP0382
4.1	1 292 278	HPF57_1206 [1 292 110– 1 293 132] (-)	*holA*	DNA polymerase III subunit delta	DNA replication	G	855	–	HP1247
4.1	1 111 855	HPF57_1042 [1 111 772– 1 112 203] (+)	*hemX*	haem interaction		C	84	–	HP0375
4.1	686 823	HPF57_0653 [686 461–688509] (+)	*mdr*	modification-specific restriction	restriction	C	363	–	no ortholog

position in F57 reference genome.

We also conducted a SNP-GWAS using another programme, pyseer, which can adjust for population structure, yielding *P*-values that were highly correlated with those obtained above (Spearman’s correlation coefficient, 0.89). Again, most *P*-values were as expected under the null hypothesis of no association, whereas there were more outlier SNPs deviating from the null hypothesis, including all the above 11 SNPs (pink dots in the Q-Q plot in Fig. S4).

In addition, after adjustment for population structure and targeting 1 345 394 unitigs (unique DNA substrings of variable length), the unitig-GWAS results showed outliers in the Q-Q plot (Fig. 2b), although the Q-Q plot seemed to deviate from the null much more than that of the SNP-GWAS (Fig. 2a) as seen in a previous study [37], possibly because of some level of population structure or more potentially highly correlated features that may not be capturing signal in the SNPs. Therefore, the *P*-values might not be directly comparable with each other. The permutation test to confirm the significance of the outlier unitigs showed that the number of unitigs showing *P*<10^−5^ under the randomly permuted phenotypes was on average 15. We thus excluded 15 out of 29 unitigs showing *P*<10^−5^, and conducted mapping to the reference genome, grouping, and annotation according to a recent study on *N. gonorrhoeae* [31], which also has a high recombination rate. We found three DNA motifs located in three different genes (bottom in Table 1).

**Fig. 3.**
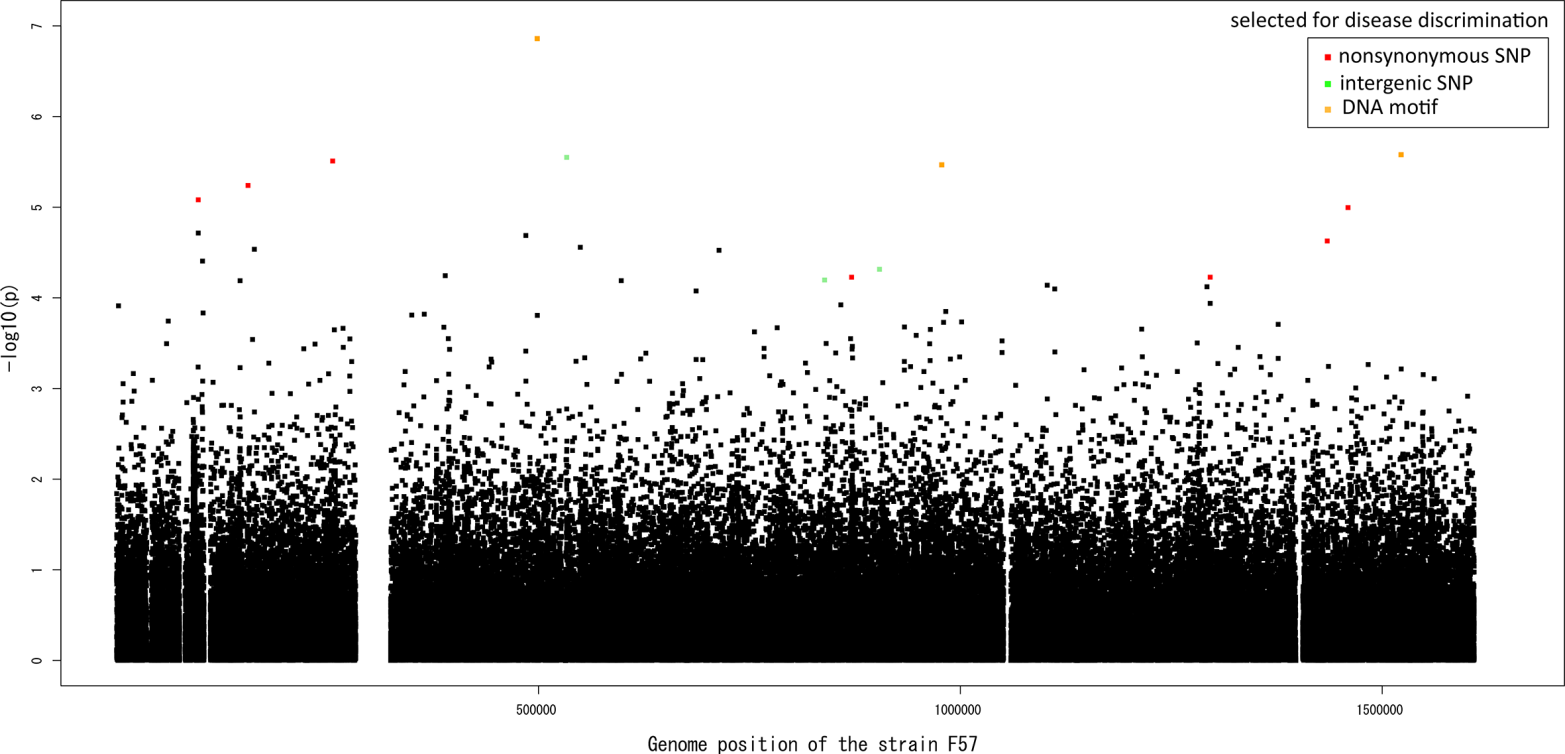
Manhattan plot summarizing the GWAS results. The nonsynonyous SNPs, integenic SNPs, and DNA motifs associated with gastric cancer are colored in red, light green, and orange, respectively. The black dots corresponds to all the other SNPs used in the SNP-GWAS.

Each SNP involved a nucleotide that showed a 12–29% frequency increase in the gastric cancer population, whereas each DNA motif showed a 19‒21 % frequency decrease or 13 % frequency increase in the gastric cancer population. The location of the 11 nucleotides and three DNA motifs associated with gastric cancer on the sequence of the Japanese reference strain F57 isolated from a gastric cancer patient is shown in Fig. 3. Clearly, there was no peak, including multiple hits, under linkage disequilibrium. The phylogenetic distribution of the 11 nucleotides and three DNA motifs associated with gastric cancer is shown as a heatmap in Fig. 1.

A random forest model using the presence or absence of the 11 nucleotides and three DNA motifs and their *P*-values (see Methods) as explanatory variables to predict the probability of a strain being isolated from a gastric cancer patient revealed that the AUC indicating discriminatory capacity was 0.88 (95 % CI, 0.82–0.94) (Fig. S5A), and false positive and false negative rate under the optimal cutoff was 0.20 and 0.20, respectively, when we conducted a cross-validation in which the first half of strains in the maximum-likelihood tree (Fig. 1) was used to train the model and the second half for prediction. We obtained similar results by two other means of cross-validation: AUC 0.86 (95 % CI, 0.78–0.93), false positive rate 0.17, and false negative rate 0.24 when the Japanese strains were used for training and the remaining strains for prediction (Fig. S5B), and AUC 0.92 (95 % CI, 0.88–0.97), false positive rate 0.16, and false negative rate 0.10 when the Vietnamese strains were used for training and the remaining strains for prediction (Fig. S5C).

Finally, we conducted a GWAS to assess the presence or absence of a specific gene among 747 genes with a minor allele frequency >5 % identified via pan-genome analysis using the panaroo pipeline [32]. Most *P*-values were as expected under the null hypothesis of no association, and there was an outlier gene deviating from the null hypothesis (Q-Q plot in Fig. S6). However, the gene was unannotated, and its frequency was 8 % in the gastric cancer population and 7 % in the duodenal ulcer population, suggesting that the difference is not biologically meaningful. Therefore, the gene was not used for further analyses.

The genes harbouring the eight non-synonymous SNPs and those closest to the three intergenic SNPs are shown in Table 1 (ordered according to the y-axis in the Q-Q plot in Fig. 2a). The genes harbouring the three DNA motifs are also shown at the bottom of Table 1. The heritability scores (h^2^) ranged from 22–33 % (median: 29%) among the SNPs and DNA motifs associated with gastric cancer and are listed in Table 1.

Each DNA motif included multiple SNPs, as shown in nucleotide sequence alignment of one of the three genes (*hsdM*, Fig. S7), reflecting the high genomic diversity of *H. pylori *. In this example, the presence or absence of this DNA motif approximately corresponds to C or T at the end of this motif, which was identified by SNP-GWAS at *P*=3.8×10^−3^. Regarding the other two DNA motifs found in unitig-GWAS, the correspondence between their presence or absence and specific amino acid changes was not clear, indicating difficulty in applying unitig-GWAS to *H. pylori *. The top-ranked SNPs in SNP-GWAS with *P*<10^−4^ were not detected in unitig-GWAS, suggesting that SNP-GWAS should mainly be used in *H. pylori *.

### Amino acid changes suggest molecular mechanisms underlying the disease phenotype

**Fig. 4.**
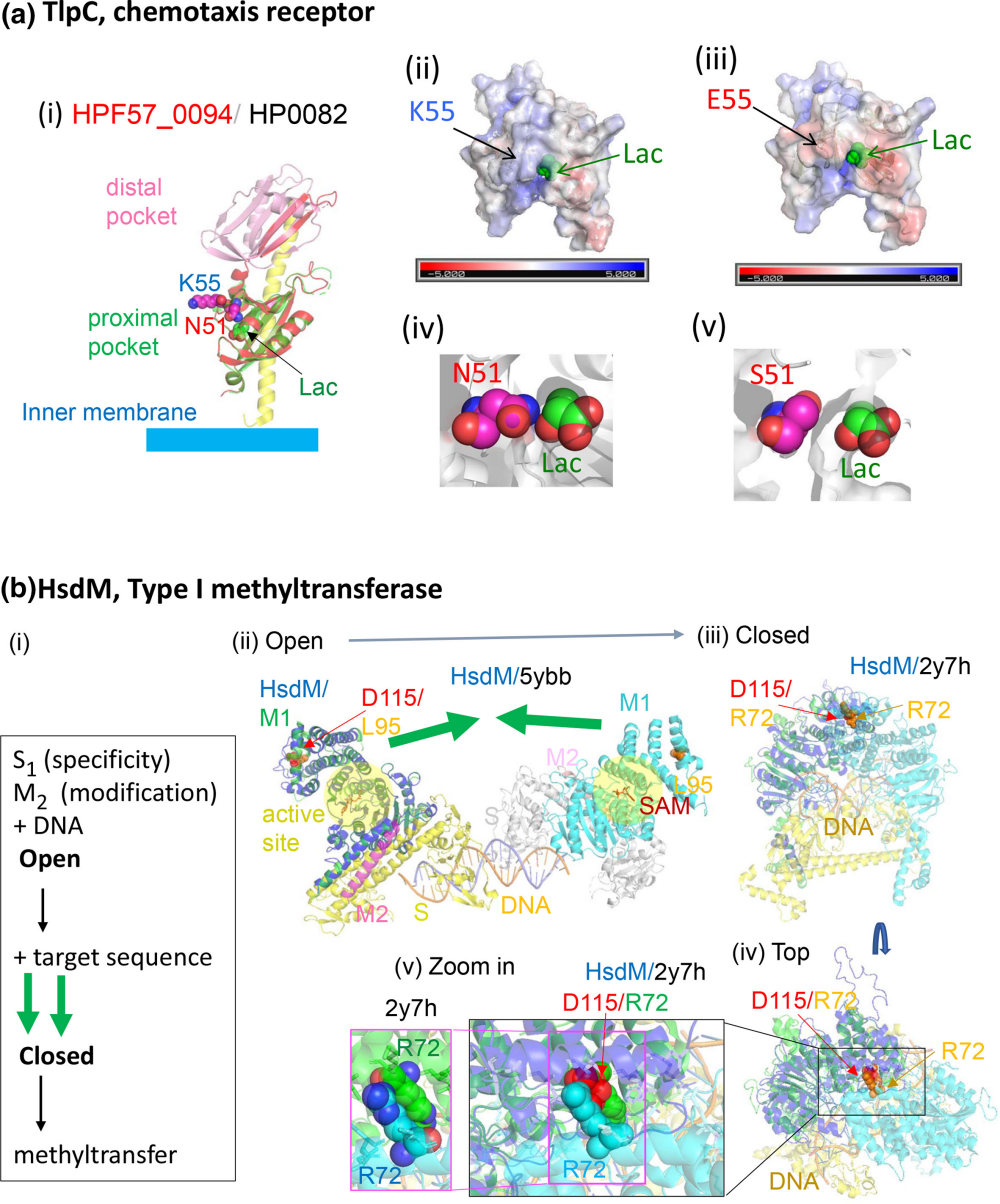
Predicted structures of proteins with discriminatory non-synonymous SNPs. (**a**) TlpC (HPF57_0094). (**i**) Model on the homolog of strain 26695 (PDB 5wbf) [38]. K55 in HPF57_0094 corresponds to E217 in HP0082, which is split into two genes in the Japanese reference strain F57. (ii)–(iii) Surface electric charges. E55 mutant protein was generated from the model by mutagenesis *in silico* (PyMOL). (iv) N51 has direct interaction with lactose. (**v**) S51 from mutagenesis (PyMOL). S51 is farther from lactose and would accommodate larger ligands. (**b**) HsdM (HPF57_0490). (**i**) Reaction steps of a Type I modification enzyme [41]. (ii) Model on 5ybb in PDB, two methyltransferase molecules, each 2M+1S, of *Caldanaerobacter subterraneus *. D115 corresponds to L95. (iii)–(**v**) Model on 2y7h in PDB, a model of EcoKI methyltransferase based on EMD-1534 [74]. D115 corresponds to R72. SNP, Single nucleotide polymorphism.

**Fig. 5.**
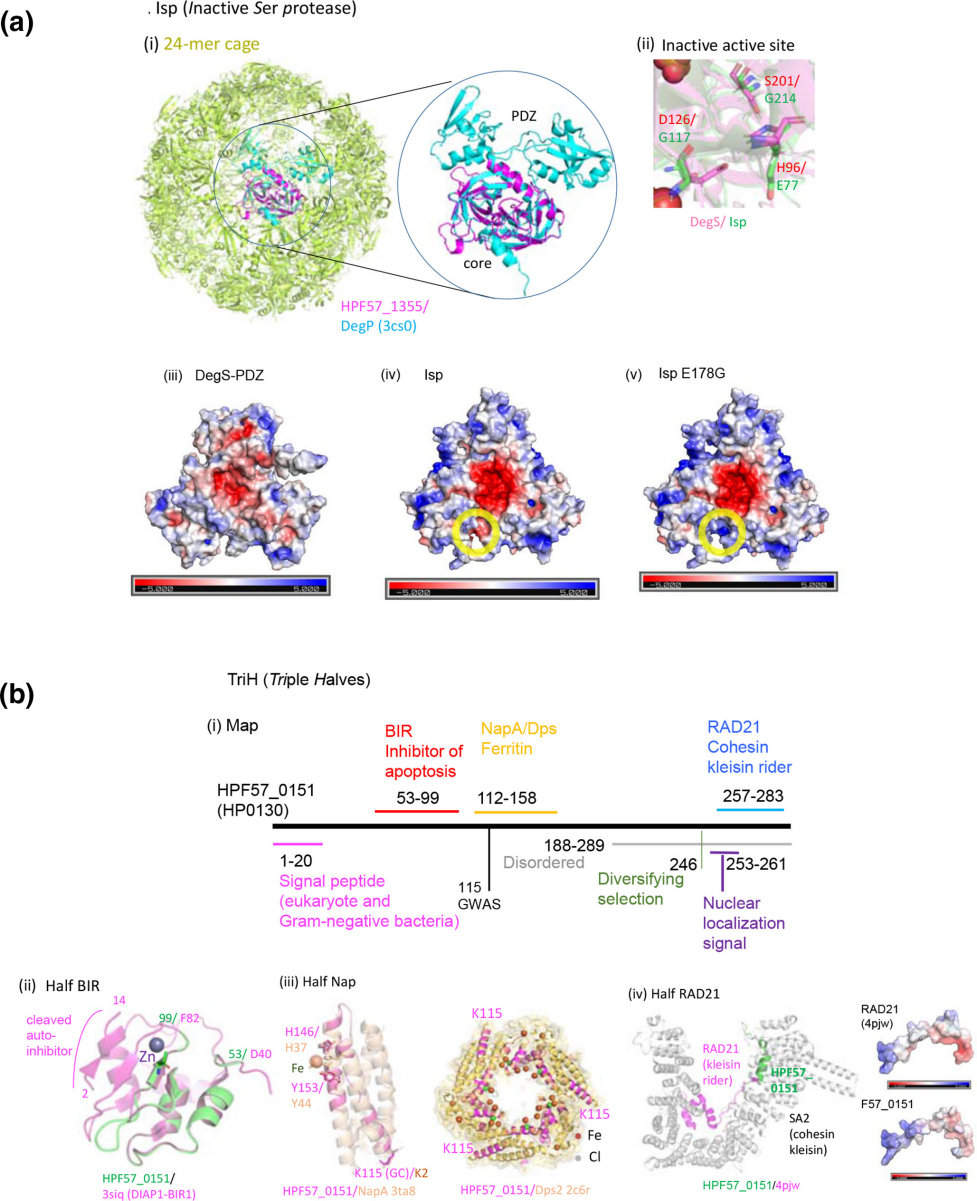
Predicted structures of three new virulence factor/oncoprotein candidates. (**a**) Isp (*i*nactive *S*er *p*rotease). (**i**) F57_1355 modelled on *E. coli* DegP. (ii) Active site of F57_1355 modelled on 3lgi.1 in PDB (*E. coli* DegS). The three amino acids (HDS triad) responsible for activity are all replaced. (iii)–(**v**) Surface electric charge distribution in *E. coli* DegS without PDZ [75] (3lgi.1 in PDB), HPF57_1355 modelled on it, and the E178G mutant generated *in silico*. (**b**) TriH, Triple halves. HPF57_0151 (HP0130). (**i**) Map. ‘Disordered’ is from UniProt. Nuclear localization signal is by cNLS Mapper. ‘Diversifying selection’ is from a previous study [49]. (ii)–(iv) Similarity to three half domains. (iii) Modelled on NapA (strain YS39, 4evd in PDB) and aligned with iron-soaked NapA (YS39, 3ta8 in PDB). Fe-interacting residues as well as the GWAS residues are in sticks. The 2c6r in PDB is Dps2 in *Deinococcus radiodurans *. Note the difference in NapA coordinates in the literature [48, 76]. (iv) HPF57_0151 modelled on PDB 4pjw (human).

We placed the amino acid changes on predicted protein 3D structures to analyse their function and biological significance. Unexpectedly, this process provided deep insights into the molecular mechanisms underlying the different diseases, as illustrated in Figs. 4 and 5, Figs. S8-S9 .

TlpC (HPF57_0094), a chemotaxis receptor (Fig. 4a (i)), has two extra-cytoplasmic ligand-binding domains. TlpC binds to lactate, which is known to promote *H. pylori* growth in the stomach, as an attractant [38]. Lactate was recently identified as a highly used molecule in the stomach, and one that varies between stomach regions [39]. The structure of its proximal pocket in the reference strain F57 was predicted to be very similar to that in the solved structure of HP0082 in the European reference strain 26 695, which contains a bound lactate. Amino acid substitution at position 55 (Table 1), corresponding to the discriminatory SNP, alters the electric charge distribution around the ligand-entry site (Fig. 4a (ii) (iii)) and may affect its ligand-binding properties, e.g., on-rate, off-rate, or affinity.Amino acid substitution at position 51 (Table 1), corresponding to another discriminatory SNP, has a direct interaction with the lactose ligand (Fig. 4a (iv)). Replacement by S51 leaves a large space, which would accommodate larger ligands. These types of changes could influence the types of responses and may in turn influence *H. pylori* growth or survival.

One GWAS motif was mapped on *hsdM* encoding a DNA methyltransferase, M subunit of a type I restriction-modification system (HPF57_0490, Fig. 4b). In addition to being responsible for modification against restriction, these methyltransferases affect gene expression [40]. When the two assemblies of the methyltransferase (2 × S1 M2) recognize their target DNA sequences, they transform from an open to a closed form [41]. Two amino acid residues of D115 on the helices of the two assemblies move a large distance to bind and thus connect the two assemblies. The motif covers right half of this helix in Fig. 4B (v) and a connected loop. A mutation in the *E. coli* homolog corresponding to the residue at position 115 and some mutations in this helix switch the enzyme from maintenance methylation mode (hemi-methylated to fully methylated DNA) to *de novo* methylation mode (unmethylated to fully methylated DNA) [42], somehow recognizing the methylation status of the target DNA sequence. This likely affects restriction attacks on incoming unmethylated DNA and on endogenous DNA upon loss of methylation. The necessity of such destructive genome maintenance is expected to differ between the stomach microbiome and the duodenal microbiome as well as between different ulcer/cancer stages.

Amino acid changes corresponding to the discriminatory SNPs included three more known virulence factors: DsbG/K disulfide bond (S-S)-forming enzyme (Fig. S8A), FecA-2 iron importer (Fig. S8B), and OmpA101 outer membrane protein (Fig. S8C). Amino acid changes with slightly higher *P*-values (up to 1.2×10^−4^, out of Table 2) included the known virulence factor CagA oncoprotein and *icfA* (Fig. S9A) encoding an enzyme involved in gastric pH homeostasis. The latter is consistent with the observation that duodenal ulcer commonly develops under a low pH condition, in contrast to gastric cancer [16].

### Novel virulence factor/oncoprotein candidates

Our GWAS identified three new virulence factor/oncoprotein candidates. The first new virulence factor candidate is Isp (*i*nactive *s*erine *p*rotease), HPF57_1355 (HP1440) (Fig. 5a). It is structurally similar to the HtrA (*h*igh-*t*emperature *r*equirement) family of serine proteases/chaperones (i), which maintains protein quality in the periplasmic or intermembrane spaces of mitochondria in animals and plants. *H. pylori* HtrA cleaves cell‐to‐cell junction factors and extracellular matrix proteins, disrupting the epithelial barrier [43]. Human HtrAs modulate mitochondrial homeostasis, cell signalling, and apoptosis, and disturbances in their action are linked to oncogenesis and neurodegeneration [44].

Although Isp and HtrA family sequences are not very similar, their structural similarity is high (QMEAN = –2.1 of 39–124 in Swiss-Prot with mitochondrial serine protease HtrA2, 5 TO 0 in PDB). The predicted structure of Isp shows its important feature, i.e., concentration of negative charges on one side of the homo-trimer joint (funnel) (iv)–(v). However, its expected active site lacks all three amino acids (HDS triad) in the active site (ii). It also lacks the arm-like PDZ domains [45] (i). It carries a signal peptide (aa 1–24, UniProt) as a bacterial DegP/Q subfamily, which includes *H. pylori* HtrA. Fig. 5a (i) shows Isp modelled on and aligned with a 24-mer cage of DegP [43].The charge in a cleft in the side of its trimer is altered by E to G substitution at position 178, corresponding to the discriminatory SNP (Fig. 5a (iv)–(v)), presumably affecting binding. We expect that this protein interferes with HtrA family proteases in human mitochondria, other bacteria, or their own (HtrA).

The second virulence factor/oncoprotein candidate is TriH (*tri*ple *h*alves) (HPF57_0151, HP0130), which carries three half pathogenicity-related domains (Fig. 5b). It carries a signal peptide (1–20) (SignalP 5.0) and is likely secreted. It also carries a strong nuclear localization signal, KPKKKRRLS (cNLS Mapper) although its nuclear localization was not experimentally verified [46]. The first half domain (aa 53–99) resembles the inhibitor of apoptosis (BIR) domain [47] (QMEAN = –1.6 in SWISS-MODEL), which inhibits a caspase (Fig. 5b (ii)) [47]; thus, this domain may interfere with apoptosis. The second domain (112–158 aa) corresponds to half of a ferritin, belonging to the Dps (DNA-protecting protein under starved conditions) family (Fig. 5b (iii)) (QMEAN = –1.5 on PDB 2c6r), which stores Fe inside 12-mer shells and protects DNA from oxidative damage. A Dps of *H. pylori *, designated NapA (neutrophil-activating protein) [48], attracts neutrophils, promotes their adhesion to endothelial cells, and induces oxygen radical production. The similar region consists of two helices out of four-helix bundles and contains only two of the four metal-binding residues [48] (Fig. 5b (iii)). Residue K115, corresponding to the discriminatory SNP, corresponds to K2 on NapA at the end of a helix. This region might modify NapA action.

The C-terminal part of TriH is disordered (UniProt) and has a site of diversifying selection (Fig. 5b) [49]. The amino acid region 257–283 is similar in sequence, structure, and electric charge to a part of RAD21/Scc1, cohesin kleisin rider (Fig. 5b (iv)) (QMEAN = –0.15). A cohesin ring coheres replicated chromosomes until its cleavage triggers metaphase-to-anaphase transition. In addition, it generates, maintains, and regulates intra-chromosomal DNA looping events. Cohesin kleisin SA2 cleaves and seals the cohesin ring under regulation by its rider, RAD21. Cohesin is among the most commonly mutated protein complexes in cancer [50], and somatic mutations and amplification of RAD21 have been reported in human tumours [51]. The N-terminus of the corresponding sequences (321–346 in RAD21) contains part of the nuclear localization signal: 317–339 in RAD21 [51] and 253–261 in TriH. We expect that this TriH domain enters nuclei and interacts with cohesin kleisin to affect cohesin action.

In addition, an amino acid change with a slightly higher *P*-value (-log_10_(*P*)=4.2) was found in HPF57_0139, HP0096, which is potentially another virulence factor/oncoprotein candidate and designated CtbP (*C-t*erminal *b*inding *p*rotein) (Fig. S9B). Zmp protease (Fig. S9D) (HPF57_1035, HP0382) acts on proteins isoprenylated at a spcific Cys but there is no such protein in the prokaryotes.

### Intergenic SNPs

One of the three intergenic SNPs was found 91 bp upstream of HPF57_0521 (corresponding to HP0490) (Fig. S10). It had the lowest *P*-value (0.000003) among the discriminatory SNPs (upper right green dot in the Q-Q plot in Fig. 2). Upstream of HP0490, there is an extended Pribnow box (tgnTAtaAT) as the –10 motif of sigma 80 preceded by periodic AT-rich sequences, although a transcription start site was not detected in previous experiments [52], likely because of the high transcription of the upstream ribosome protein gene, HP0491. In a predicted secondary structure (M-fold) of the expected transcript, the discriminatory SNP was located at a loop-stem boundary, presumably slightly affecting interaction with a protein or an RNA. Use of the sub-optimal UUG start codon instead of AUG and presence of antisense RNA (HPnc2800) suggest tight regulation of HP0490. The HP0490 gene (*kch, trkA*) encodes a K^+^ channel protein regulating K^+^ conductance across the membrane (UniProt) and is essential for *H. pylori* colonization of the murine stomach [53]. The SNP might modulate the expression of K^+^ conductance for persistence in the gastric/duodenal environment.

The second intergenic SNP is present in the promoter region (–29 bp) of the omega subunit of RNA polymerase (HPF57_0798). (In *H. pylori *, the upstream promoter element is characterized by an AT-rich sequence [34]). *E. coli* omega binds to ppGpp alarmon [54] in stringent response [55] although its N-terminal MAR motif for ppGpp-binding is not conserved in *H. pylori* [55]. *E. coli* omega affects transcription of prophage genes not bound by the H-NS silencer [56]. Stringent responce in *H. pylori* takes place upon acid exposure. Prophage action may differ between duodenal ulcer and gastric cancer as mentioned above for restriction-modification systems.

The third intergenic SNP at 904 207 in F57 lies 14 bp downstream of an operon-like gene cluster for vitamin B1 (thiamine diphosphate) synthesis, *thiM-thiD-thiE* (HPF57_0867- HPF57_0865, HP0845-HP0843). It disrupts the stem GGAAUU/CCUUAA of the first stem-loop and might affect the expression of these genes and vitamin B1 synthesis. Thiamine derivatives bind directly to mRNA to regulate gene expression (riboswitch) in bacteria [57]. Vitamin B1 availability may differ between the stomach and duodenum and between cancer cells and other cells. *H. pylori* may even supply vitamin B1 to human cells to affect their growth as the microbiome contributes to vitamin metabolism [58].

## Discussion

This was a GWAS to reveal gastric cancer-related genetic features by focusing on the highest-risk *H. pylori* population of gastric cancer and to utilize the dataset of *H. pylori* strains isolated from gastric cancer and duodenal ulcer patients. Generally, it is difficult to obtain a large number of these samples because as the cancer stage progresses, it becomes more difficult to isolate *H. pylori *. Of note, it is difficult to obtain not only appropriate samples, but also consistent and reliable follow up data. The dataset covers more than 100 strains from gastric cancer patients, as well as those from duodenal ulcer patients, which enabled the GWAS. However, we should keep in mind that the sample size is still smaller than that of GWASs in other bacterial species that utilized thousands of genome sequences [30, 59, 60]. Accordingly, the statistical power was insufficient to judge whether each discriminatory SNP indeed has a significant effect. When we conducted a standard multiple-testing correction, the false discovery rate was at least 0.24 for the most significant SNP. Further studies, if possible, with larger sample sizes, are thus warranted to test the effect of each SNP.

Thus, we rather examined the combined effect of all SNPs and DNA motifs deviating from the null hypothesis in the Q-Q plot. To this end, we used a random forest model to predict gastric cancer and duodenal ulcer *H. pylori* strains using information of the presence or absence of the top-hit SNPs and DNA motifs, which worked well (with AUC >0.85 in all three cross-validations). Although cross-validation was conducted because of the small sample size, further studies are warranted to prepare another independent dataset of *H. pylori* isolated from gastric cancer and duodenal ulcer patients and utilize it to statistically validate whether discrimination using the SNPs and DNA motifs works well in another external population.

In the present study, duodenal ulcer was treated as the ‘control’ phenotype, given the importance of early-stage discrimination between gastric cancer and duodenal ulcer as the two major disease progression pathways and the difference in treatment guidelines between them. However, evaluation of how to better predict one of them is another problem, which requires another control group and comparison between control vs gastric cancer and control vs duodenal ulcer. However, there is no appropriate control dataset of *H. pylori* strains isolated from healthy controls or slight NAG, which was determined to have not started progression toward cancer. Furthermore, our *H. pylori* strains from the duodenal ulcer patients were obtained from their stomach instead of their duodenum.

Correct disulﬁde bond (S-S) formation is critical in the folding of many secretory and membrane proteins in bacteria, including toxins, adherence factors, and components of secretory systems. The highly variable thiol:disulﬁde oxidoreductases of the Dsb (*d*i*s*ulﬁde *b*ond) family catalyse this step in the periplasm in Gram-negative bacteria. DsbG/K of *H. pylori* (Fig. S8A), a homolog of DsbG of *E. coli*, is secreted and affects the stomach epithelium [61] and enables colonization [62]. It can interact with and refold reduced HcpE (HP0235) [63]. DsbG/K acts on HopQ and helps HopQ-CEACAM interaction for the delivery of CagA in which a discriminatory SNP was found. Therefore, the loci identified by our GWAS are not only multifaceted, but can interact with and affect each other.

Another discriminatory SNP was identified in FecA-2 (Fig. S8B) involved in iron uptake. Iron uptake and metabolism are central to *H. pylori* survival and host interaction. Current evidence indicates that *H. pylori* infection is related to an increased likelihood of depleted iron storage (iron deficiency anaemia) [64]. Human iron metabolism is relevant to carcinogenesis [65], and iron deficiency increases *H. pylori* virulence and the risk of gastric cancer [64]. A large part of the *fecA-2* gene is deleted in hspAmerind strains. Mutations in *fecA-2* have been observed after *H. pylori* diversification in the Mongolian gerbil [66].

The discriminatory SNP with the lowest *P*-value was located upstream of *kch* (*trkA*) (Fig. S10), encoding a K^+^ channel protein essential for *H. pylori* colonization of the murine stomach. In human epithelial cells, various K^+^ channels are expressed, allowing adaptation to different needs in different organs [67]. In the human gastric mucosa, K^+^ channel function is a prerequisite for acid secretion by parietal cells. In epithelial cells of the small intestine, K^+^ channels provide the driving force for electrogenic transport across the plasma membrane, and they are involved in cell volume regulation. Similarly, *H. pylori* may express this K^+^ channel in different ways for different needs in the two organs.

In addition to known virulence factors, our GWAS revealed three hitherto unrecognized virulence factor candidates: Isp (*i*nactive *s*erine *p*rotease), TriH (*tri*ple *h*alves) (Fig. 5) and CtbP (Fig. S9B). A likely common mechanism is interference with specific host proteins, a strategy shown for CagA, although there is still an element of competition for the small molecule NAD. They resemble several tumour virus oncoproteins, such as E1A of adenovirus and Tax of HTLV, which take over, by protein-protein interaction, the human cell protein network for survival of the infected cells [68]. *H. pylori *, an oncogenic bacterium, may use the same strategy as tumour viruses in addition to competition with human cells over small molecules through transporters.

There is growing evidence that synonymous SNPs can affect gene expression, protein folding, and ultimately the fitness of an organism [69–71]. However, we are aware that synonymous SNPs are more likely to be false positives than nonsynonymous ones, and thus excluded all the nine synonymous SNPs from further analysis based on the permutation test to confirm significance of the outlier SNPs. Nonetheless, we found interesting genes with a synonymous SNP (Table 2), some of which including known virulence factors are described in Fig. S11.

A previous GWAS comparing *H. pylori* from patients diagnosed with NAG and gastric cancer with a focus on the hpEurope populationrevealed 32 gastric cancer-associated loci. These genes mostly belonged to the *cag* pathogenicity island (PAI) and encoded outer membrane proteins, such as *babA*. In our GWAS, focusing on the hspEAsia population, we found none of these previously reported gastric cancer-associated loci. This discrepancy is likely due to two major differences. First, the preceding study compared gastric cancer and gastritis, while we compared gastric cancer and duodenal ulcer. Gastric cancer develops from gastritis, and the two diverge at an early stage. Second, the hpEurope and hspEAsia populations are genetically different. In particular, hpEurope includes 50–60% *cag* PAI-positive strains, whereas hspEAsia strains are nearly all *cag* PAI-positive [72]. The organization of *babABC* loci/alleles as well as other outer membrane proteins is quite different in hpEurope and hspEAsia strains.

The set of discriminatory SNPs and DNA motifs identified in this study will be potentially applicable to personalized risk stratification in clinical settings for early-stage discrimination between gastric cancer and duodenal ulcer and for the selection of appropriate treatments. A recent study developed a high-throughput multiple allele detection assay [73]. Incorporation of the discriminatory SNPs and DNA motifs into such a technique will assist clinicians in diagnosis and clinical decision making.

In conclusion, our study revealed multifaceted pgenetic features of *H. pylori* associated with the pathogenesis of gastric cancer as compared to duodenal ulcer, and demonstrated the effectiveness of GWAS followed by prediction in distinguishing these *H. pylori *-related diseases, although the individual effect of each discriminatory genetic variation was not significant despite using the largest-to-date, but still limited sample size dataset. Although application of the prediction markers in distinguishing these *H. pylori *-related diseases in clinical settings requires more validation, our analysis provided a basis for it and insights into the pathogenesis of gastric cancer.

## Supplementary Data

Supplementary material 1

Supplementary material 2

Supplementary material 3
