# Corrigendum: ‘Genome-wide association study of gastric cancer- and duodenal ulcer-derived *Helicobacter pylori* strains reveals discriminatory genetic variations and novel oncoprotein candidates’

**DOI:** 10.1099/mgen.0.001062

**Published:** 2023-07-06

**Authors:** Vo Phuoc Tuan, Koji Yahara, Ho Dang Quy Dung, Tran Thanh Binh, Pham Huu Tung, Tran Dinh Tri, Ngo Phuong Minh Thuan, Vu Van Khien, Tran Thi Huyen Trang, Bui Hoang Phuc, Evariste Tshibangu-Kabamba, Takashi Matsumoto, Junko Akada, Rumiko Suzuki, Tadayoshi Okimoto, Masaaki Kodama, Kazunari Murakami, Hirokazu Yano, Masaki Fukuyo, Noriko Takahashi, Mototsugu Kato, Shin Nishiumi, Takashi Azuma, Yoshitoshi Ogura, Tetsuya Hayashi, Atsushi Toyoda, Ichizo Kobayashi, Yoshio Yamaoka

**Affiliations:** ^1^​ Department of Endoscopy, Cho Ray Hospital, Ho Chi Minh, Vietnam; ^2^​ Department of Environmental and Preventive Medicine, Oita University Faculty of Medicine, Oita, Japan; ^3^​ Antimicrobial Resistance Research Center, National Institute of Infectious Diseases, Tokyo, Japan; ^4^​ Department of GI Endoscopy, 108 Central Hospital, Hanoi, Vietnam; ^5^​ Department of Molecular Biology, 108 Military Central Hospital, Hanoi, Vietnam; ^6^​ Department of Microbiology, Cho Ray Hospital, Ho Chi Minh, Vietnam; ^7^​ Department of Gastroenterology, Oita University Faculty of Medicine, Yufu, Oita, Japan; ^8^​ Department of Computational Biology and Medical Sciences, Graduate School of Frontier Sciences, University of Tokyo, Tokyo, Japan; ^9^​ Institute of Medical Science, University of Tokyo, Tokyo, Japan; ^10^​ Graduate School of Life Sciences, Tohoku University, Sendai, Japan; ^11^​ Department of Molecular Oncology, Chiba University, Chiba, Japan; ^12^​ Department of Infectious Diseases, Kyorin University School of Medicine, Mitaka City, Tokyo, Japan; ^13^​ Division of Endoscopy, Hokkaido University Hospital, Sapporo, Hokkaido, Japan; ^14^​ Department of Gastroenterology, National Hospital Organization Hakodate Hospital, Hakodate, Hokkaido, Japan; ^15^​ Department of Gastroenterology, Graduate School of Medicine, Kobe University, Chuou-ku, Kobe, Hyogo, Japan; ^16^​ Department of Omics Medicine, Hyogo College of Medicine, Hyogo, Japan; ^17^​ Department of Bacteriology, Faculty of Medical Sciences, Kyushu University, Fukuoka, Japan; ^18^​ Division of Microbiology, Department of Infectious Medicine, Kurume University School of Medicine, Kurume, Fukuoka, Japan; ^19^​ Advanced Genomics Center, National Institute of Genetics, Shizuoka, Japan; ^20^​ Research Center for Micro-Nano Technology, Hosei University, Tokyo, Japan; ^21^​ Department of Medicine, Gastroenterology Section, Baylor College of Medicine, Houston, TX, USA

## Full-Text

In the published version of this article, two errors of locus tags in Table 2 have been corrected as follows: HP0915 has been changed to HP0776, and HP_0915 has been changed to HP0915.

The authors apologise for any inconvenience caused.

