# Supplementary material for: Genome-wide association study of gastric cancer- and duodenal ulcer-derived Helicobacter pylori strains reveals discriminatory genetic variations and novel oncoprotein candidates
Source: Microb Genom. 2021 Nov 30;7(11):000680. doi: 10.1099/mgen.0.000680 (PMC8743543; doi:10.1099/mgen.0.000680)
Supplement: Supplementary material 1 [file mgen-7-0680-s001.pdf]

Supplementary Figures

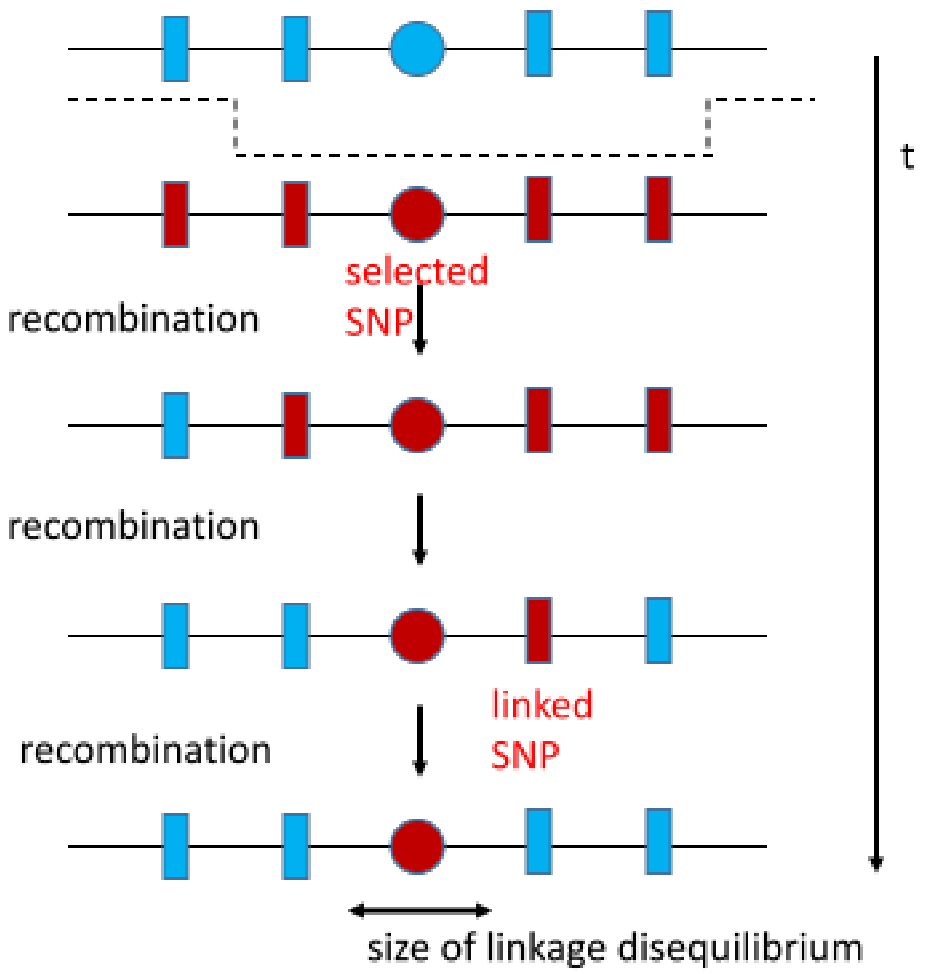

S1 Figure. Schematic depiction of the decrease of linkage disequilibrium after repeated recombination events.

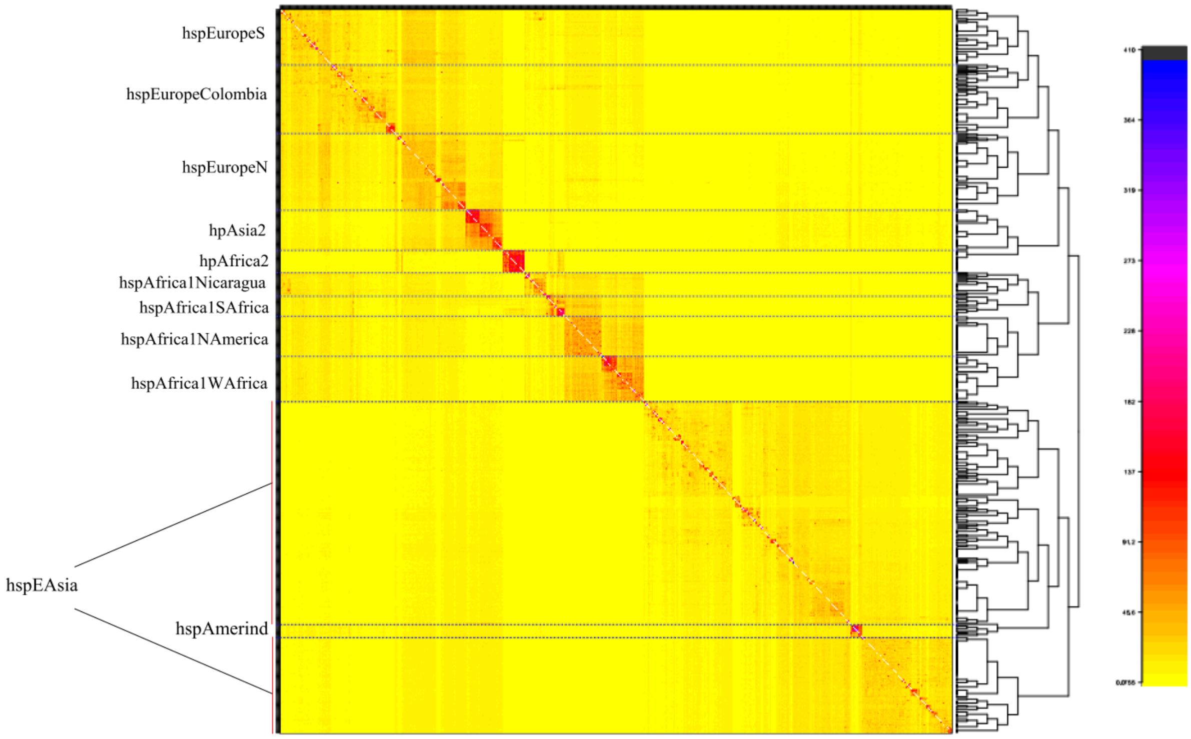

S2 Figure. Population structure of 614 global *H. pylori* strains. The color of each cell of the co-ancestry matrix indicates the expected number of DNA chunks imported from a donor genome (column) to a recipient genome (row). Dashed lines separate the different populations. Strains belonging to hspEAsia are indicated by the red vertical lines. Detailed information on the *H. pylori* strains used in this analysis is shown in Table S1.

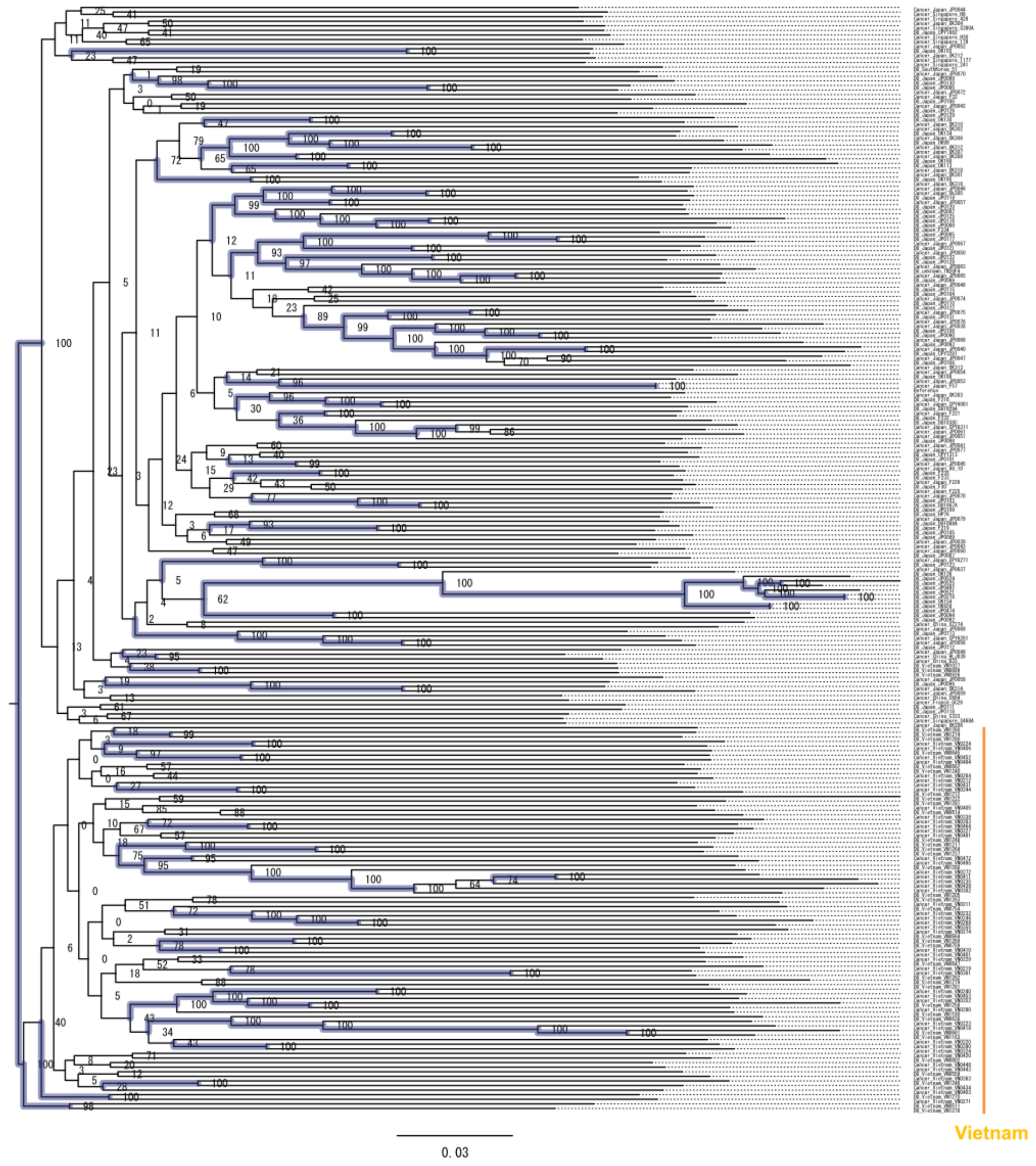

S3 Figure. Maximum-likelihood tree with bootstrap values. All nodes and branches with bootstrap values with > 90% are colored in purple.

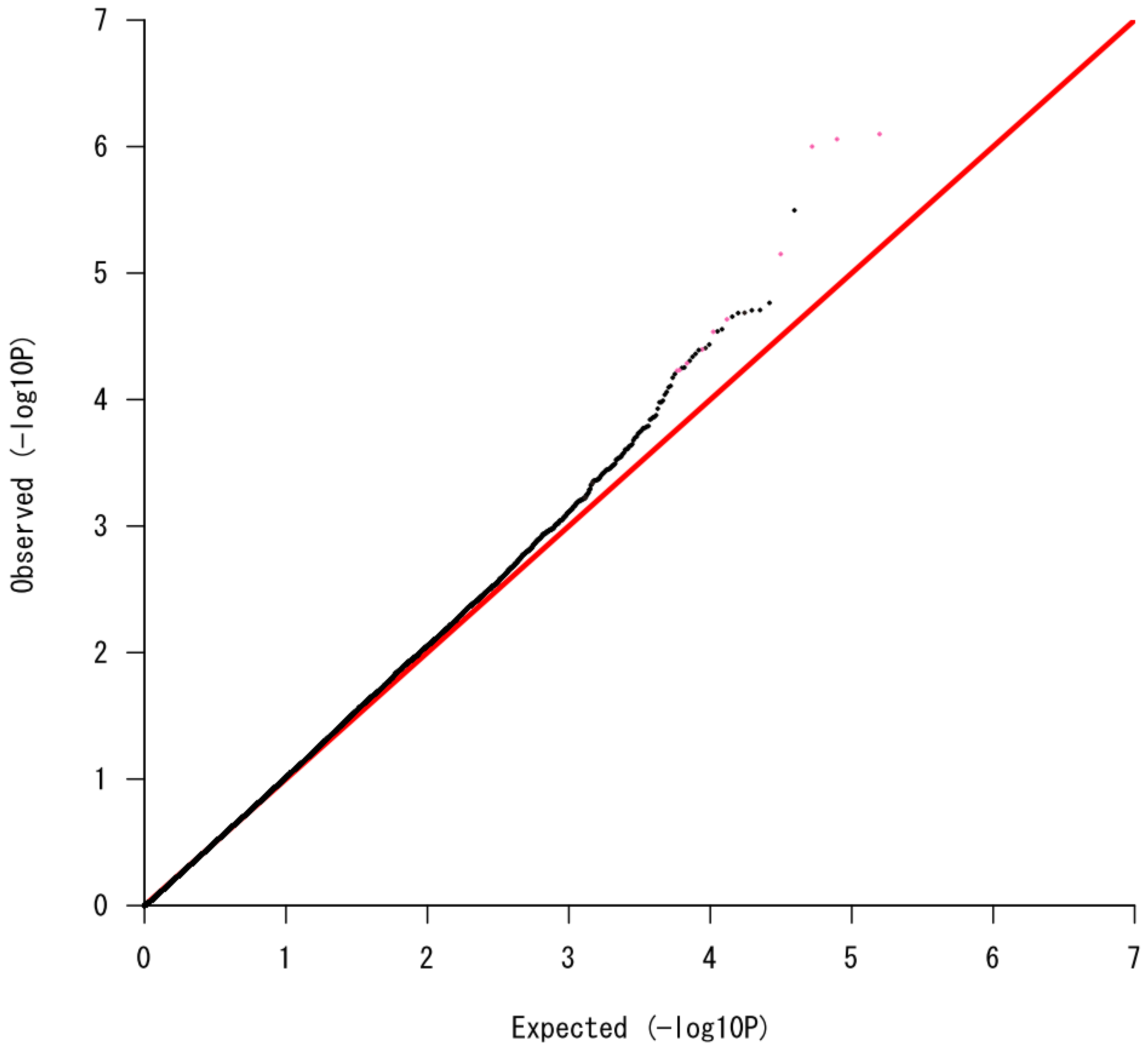

S4 Figure. Q-Q plot to assess GWAS results using FaST-LMM implemented in pyseer.

The 11 SNPs identified in Figure 2A are indicated in pink.

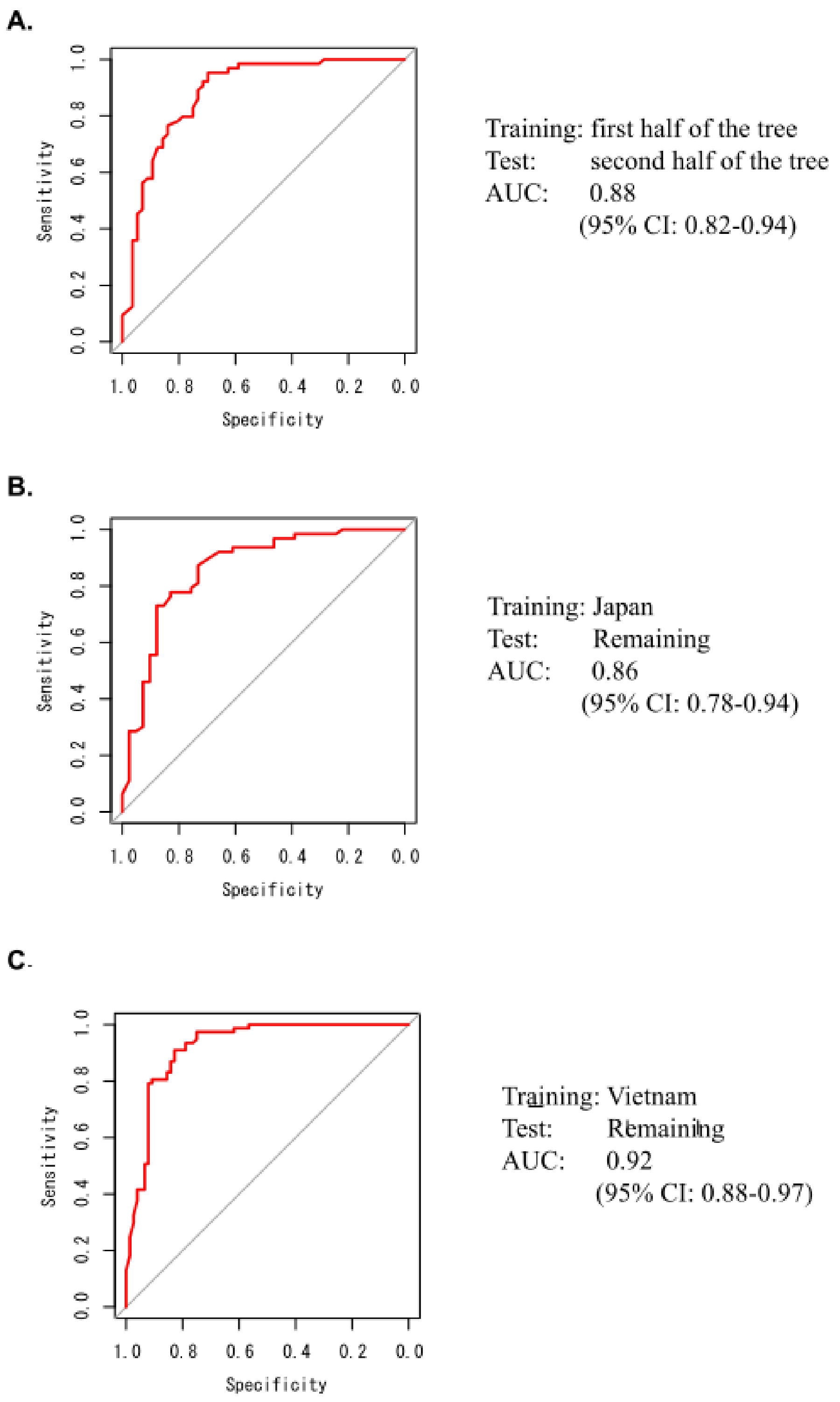

S5 Figure. ROC curves representing performance of the discrimination. Sensitivity (y-axis) and specificity (x-axis) were calculated using three means of 2-fold cross validations and cutoffs of the predicted probability of being isolated from a gastric cancer patient for an strain.

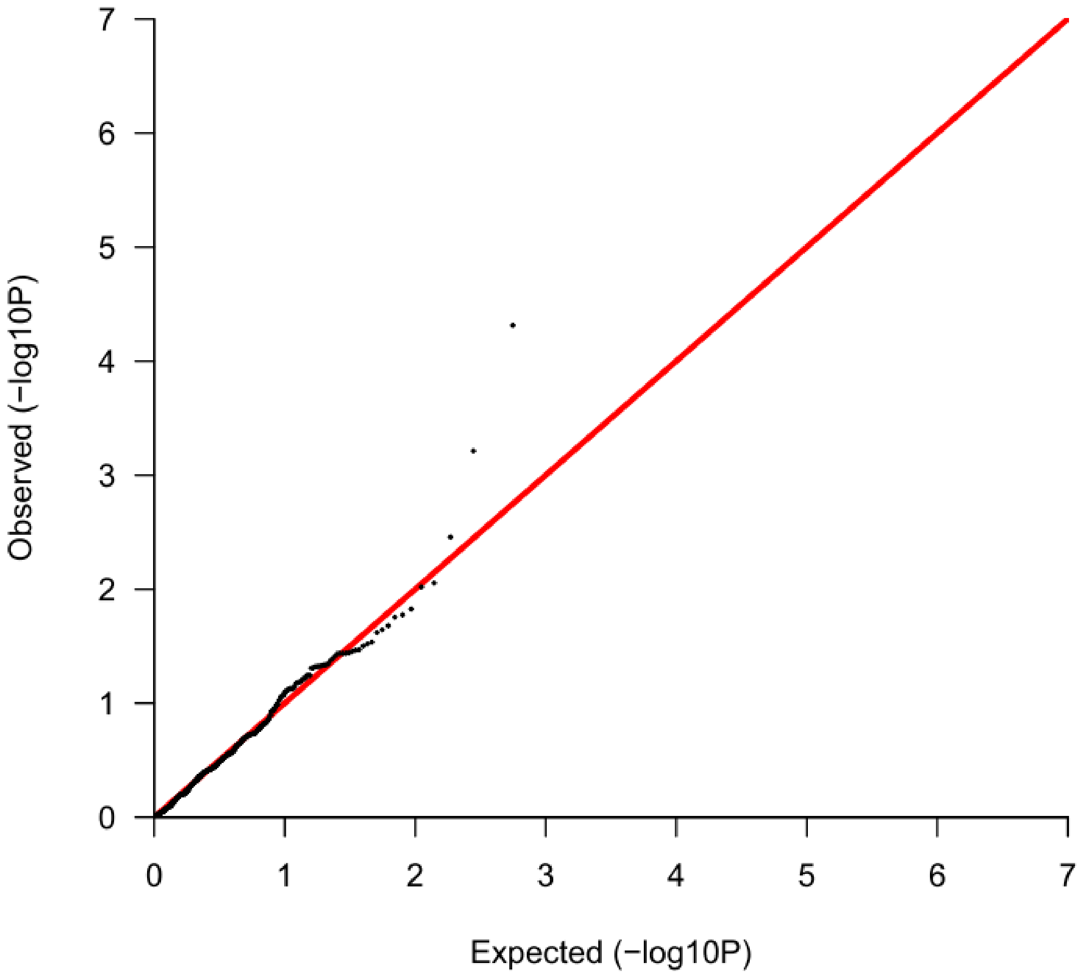

S6 Figure. Q-Q plot to assess GWAS results regarding the presence or absence of a specific gene.

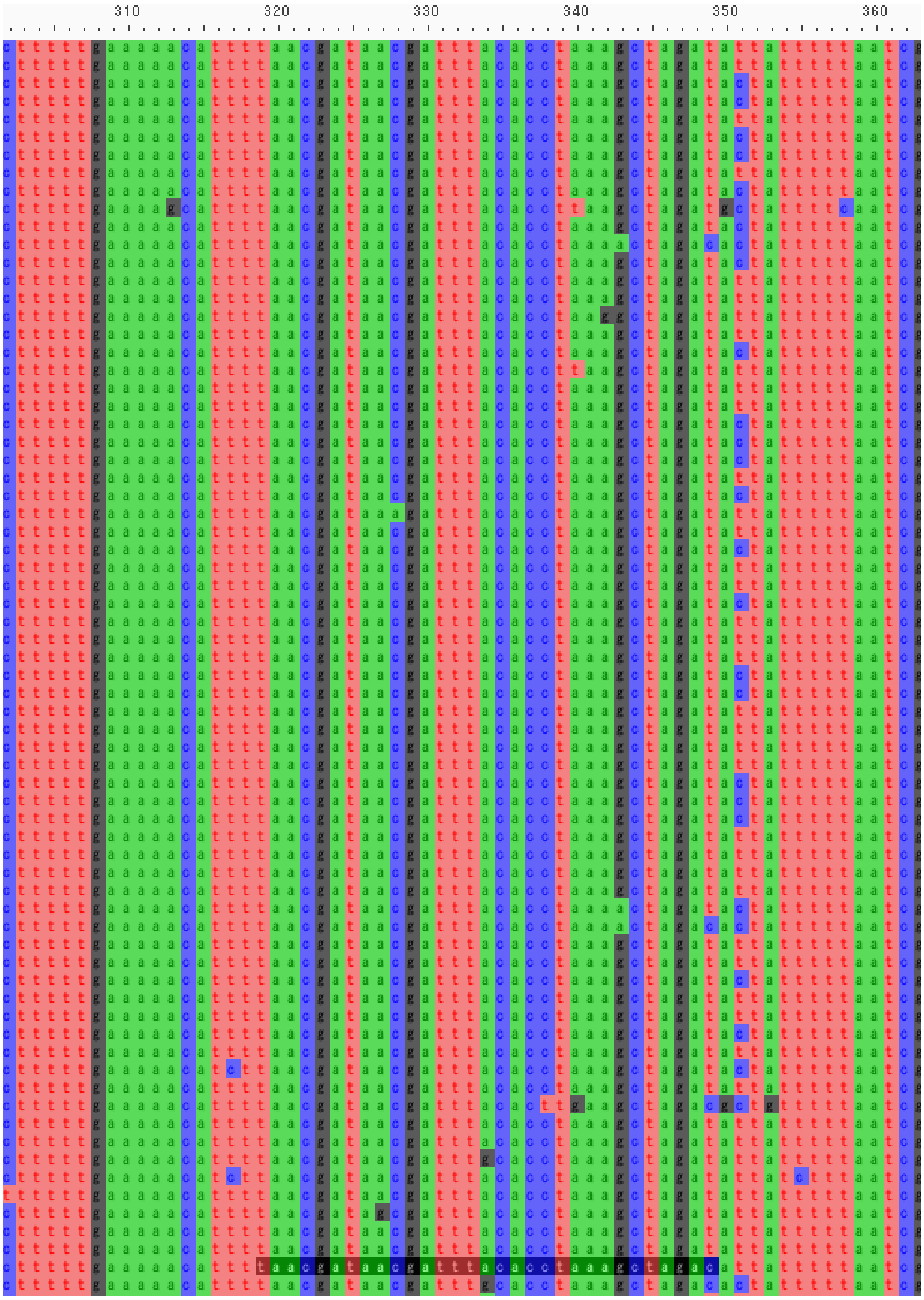

S7 Figure. Nucleotide sequence alignment of a part of *hsdM* showing the unitig and multiple SNPs included in it. The unitig (Table 1) is shaded at the bottom.

A. DsbG/K

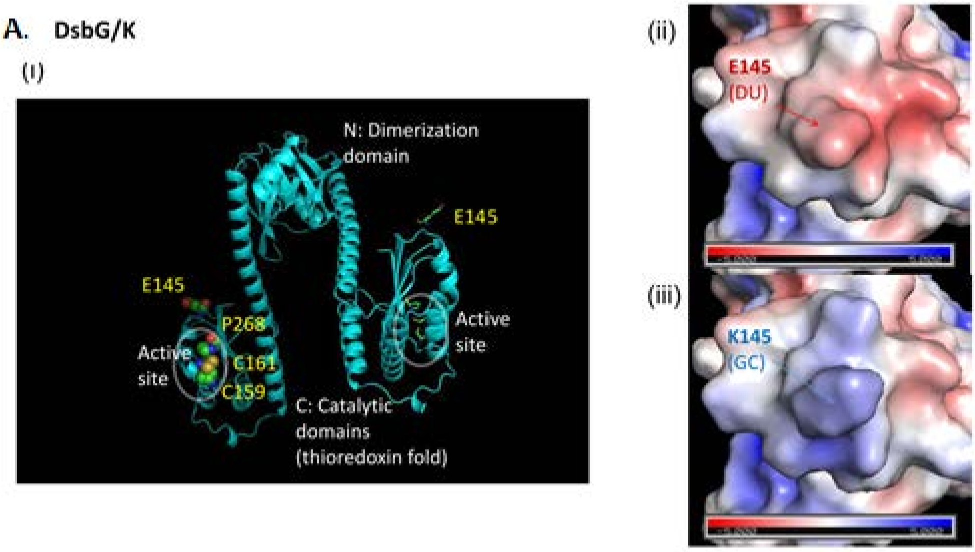

B. FecA2, iron (III) dicitrate importer

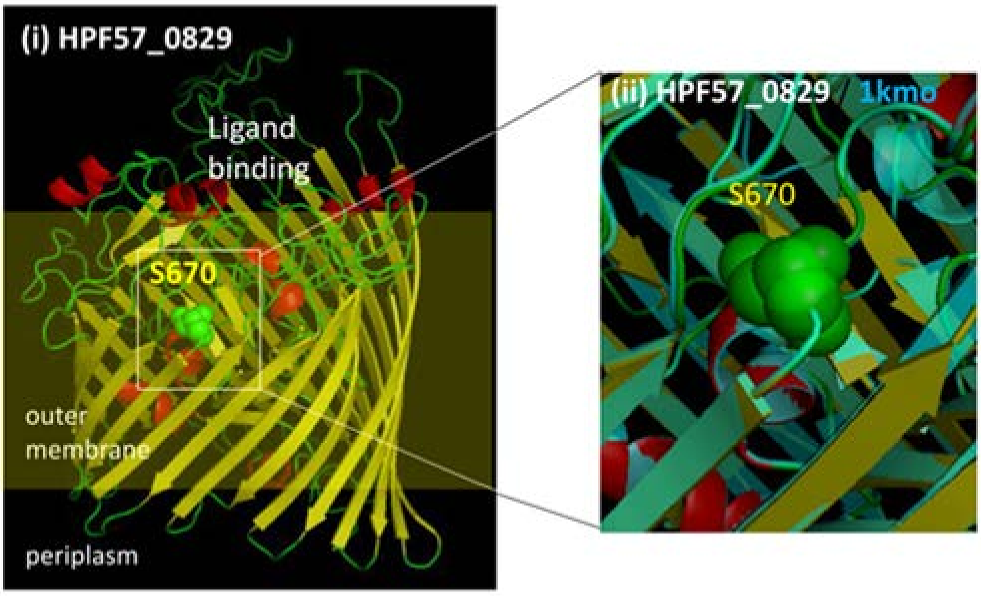

C. OmpA101

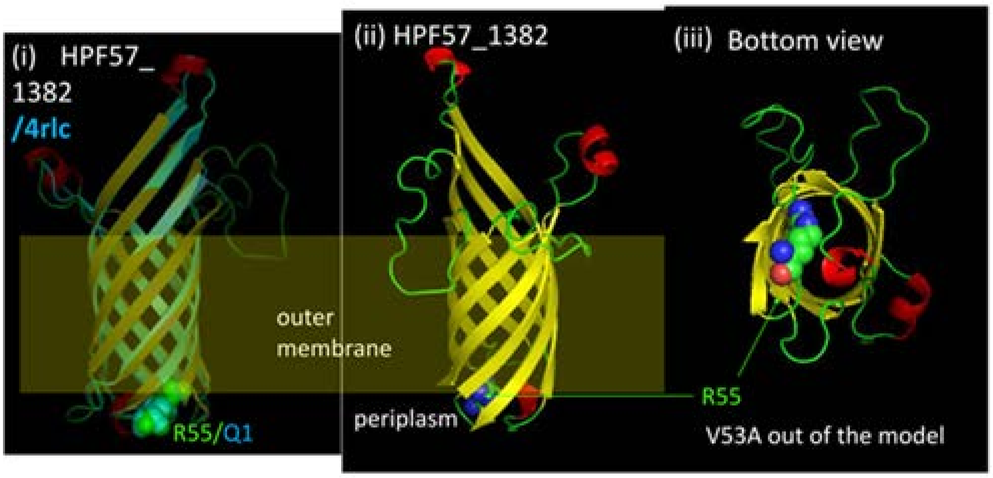

S8 Figure. Proteins with amino-acid changes at a discriminatory SNP.

A. DsbG/K. **(i)** HPF57_0250 was modeled on HP0231 with 99% amino-acid identity (3tdg.1.A in PDB). The C-terminal domain (residues 132–265) has the thioredoxin-like fold with the catalytic CXXC motif and cis-Pro loop. The GWAS SNP site E145 (duodenal ulcer) lies at one side of the C-terminal domain distant from the CXXC motif. **(ii, iii)** Its mutation to K145 drastically changes the surface electric charge distribution and likely affects its function as a redox enzyme and its interaction with other components.

B. FecA-2, a TonB-dependent outer membrane iron (III) dicitrate transporter. HPF57_0829 was modeled on 1kmo of PDB (FecA of *Escherichia coli*). S670 is predicted to be in a loop right above the wall of the barrel. The same location was found with models on 4aip and 4aiq (FrpB of *Neisseria meningitides*).

C. OmpA101. HPF57_1382 was well modeled on *E. coli* OmpA structures and PDB 4rlc (N-terminal domain of *Pseudomonas aeruginosa* OprF) ((i)–(iii)). They are assembled into an 8-stranded β-barrel by the Bam machinery and may function in the uptake of small molecules (Reusch 2012). V53A is on the second amino-acid residue from R55 at the N-terminus of the model, which lies in a loop at the bottom of the barrel. It is located after a predicted signal peptide (1–22) (SignalP-5.0). Based on the length from the barrel model, V53A corresponds

to Q20 of *E. coli* OmpA, which is at the end of its signal sequence. V53A might affect ligand exit or own translocation through inner membrane.

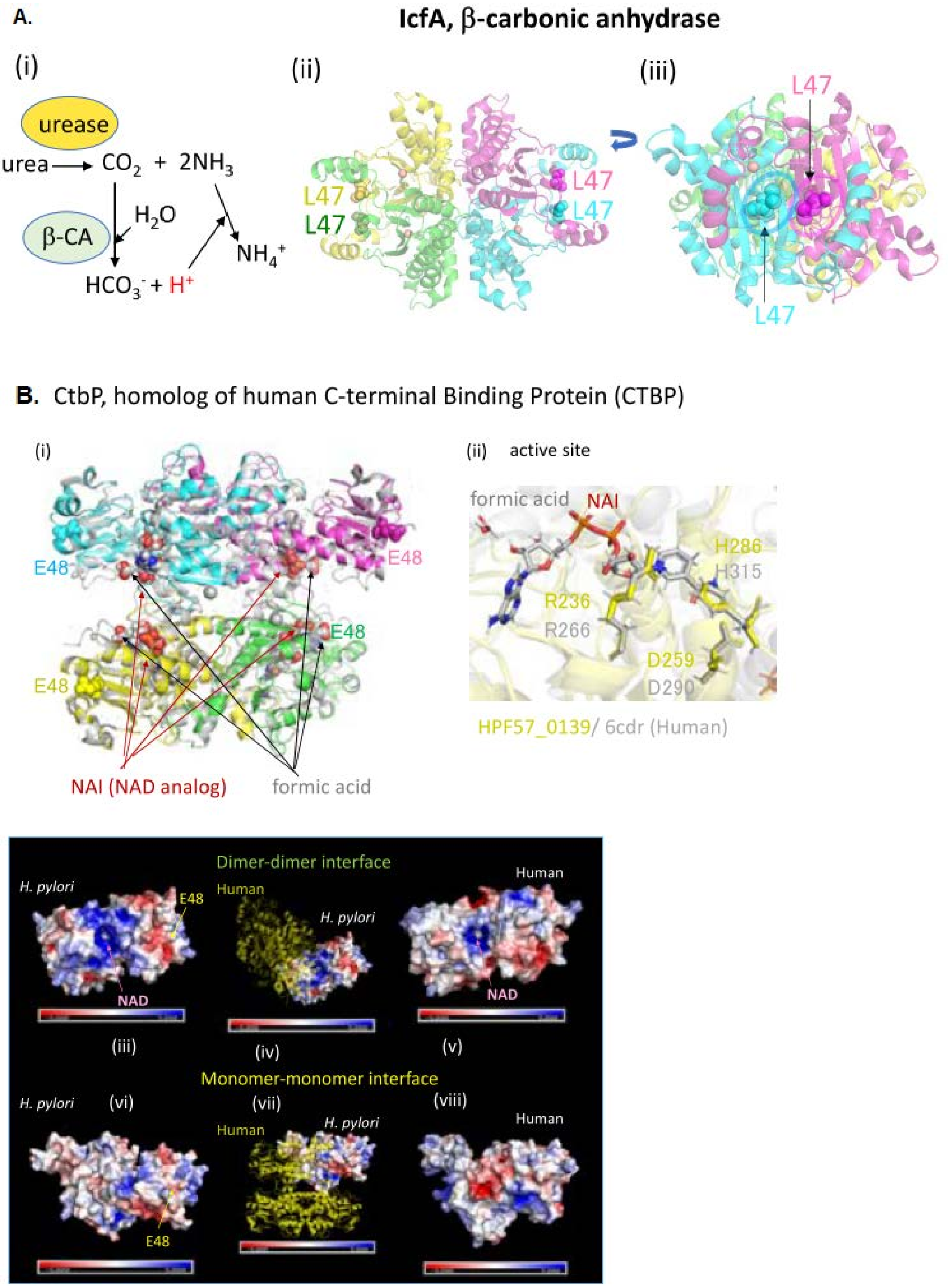
A.
IcfA, β-carbonic anhydrase
(i)
urease
urea → CO2 + 2NH3
β-CA
H2O
NH4+
HCO3- + H+
(ii)
L47
L47
L47
L47
(iii)
L47
L47
B. CtbP, homolog of human C-terminal Binding Protein (CTBP)
(i)
E48
E48
E48
E48
NAI (NAD analog)
formic acid
(ii) active site
formic acid
NAI
H286
H315
R236
R266
D259
D290
HPF57_0139/ 6cdr (Human)
Dimer-dimer interface
H. pylori
E48
NAD
Human
H. pylori
Human
NAD
(iii)
(iv)
(v)
Monomer-monomer interface
H. pylori
(vi)
(vii)
(viii)
Human
H. pylori
Human
E48

C. CagA

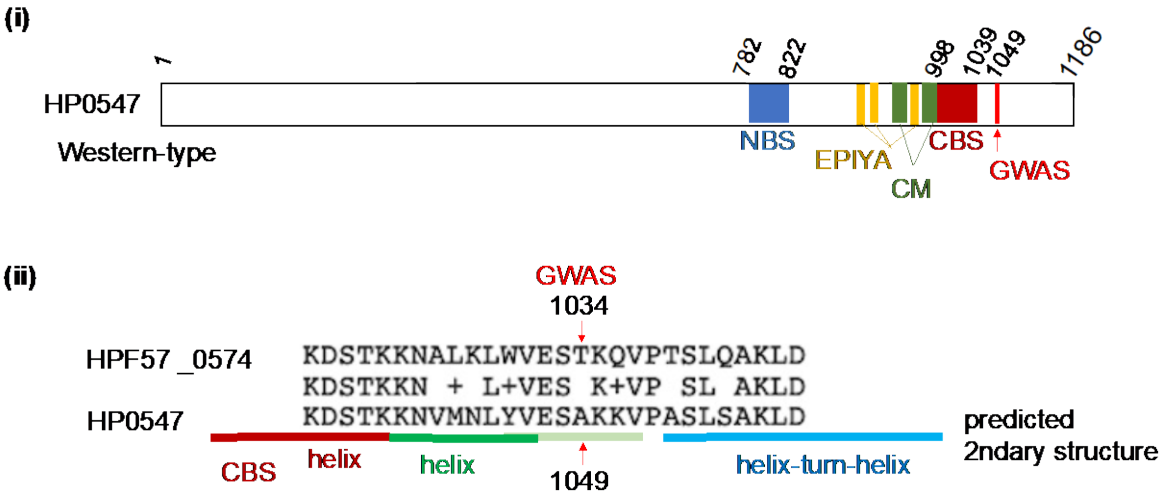

D. Zmp

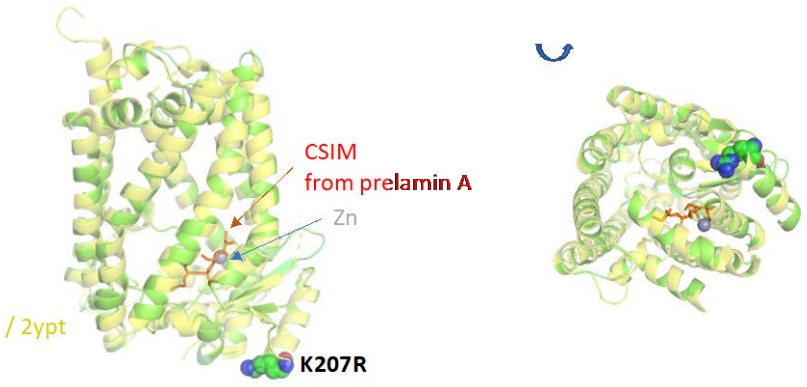

S9 Figure. Proteins with amino-acid changes with slightly higher P-values. A. IcfA (HPF57_0004). (i) Acid acclimation in the cytoplasm together with urease. (ii)–(iii) HPF57_0004 on *E. coli* homolog (PDB 1i6p). The α family carbonic anhydrase in the periplasm processes CO_2 produced from urea by urease and is essential for acid acclimation (Supuran and Capasso 2016) and involved in gastric pH homeostasis. The β family of carbonic anhydrases, including IcfA, likely plays the same role in the cytoplasm (Nishimori, et al. 2007). Residue L47, corresponding to the discriminatory SNP, forms a pair at the dimerization joint. We expect that this residue affects subunit interaction to regulate the

reaction speed of this very fast-acting enzyme. It could be related to the elevated acid secretion in duodenal ulcer and its suppression during gastric cancer progression.

B. HPF57_0139, HP0096, designated CtbP (*C*-*t*erminal *b*inding *p*rotein). (i) Model on human CtBP1 (PDB 6cdr). (ii) Similarity of their active sites. (iii) Subunit interaction and surface electric charges in a model on 3kb6 (*Aquifex aeolicus* homolog) and CtBP1. This protein is similar to the human "C-terminal of adenovirus E1A" binding protein 1 (CtBP1) (34% amino acid sequence identity in BLAST) and CtBP2 (32%) (Bellesis, et al. 2018) in sequence and in structure (GMQE = 0.69 and QMEAN = –1.99 on CtBP1 (6cdr in PDB) and GMQE = 0.66 and QMEAN = –2.42 on CtBP2 (4lcj in PDB) in SWISS-MODEL). This family (NAD-dependent D-isomer specific 2-hydroxyacid dehydrogenase, CH-OH + NAD => C=O + NADH) is prevalent. Human CtBPs are NAD-sensing transcriptional co-regulators that bind to transcription factors and recruit chromatin-remodeling enzymes to gene promoters (Chinnadurai 2009). Their dehydrogenase activity with NAD as the acceptor is used to monitor intracellular NADH/NAD concentrations (or the energy state in a sense). Both CtBPs have been implicated in repression of the epithelial phenotype and of apoptotic pathways, and in cancer progression (Chinnadurai 2009). The *H. pylori* homolog has a more basic charge around the NAD-binding site (Figure 5A (iii) vs. (v)) and a stronger dimer-dimer interaction (Bellesis, et al. 2018). *H. pylori* CtbP might interfere with NAD sensing and/or assembly of human CtBPs and affect their NAD-based transcription regulation and cell death/differentiation. *H. pylori* may inject its CtbP into the host cell to hack it at the CtBP hubs of a protein-protein network, just as adenovirus uses E1A (King, et al. 2018). V65 in CtBP1, corresponding to GWAS amino acid E48 in *H. pylori* CtbP, is located at the N-terminal region (1–70), which likely binds to GLIS2 (GLI-similar 2), a Krüppel-like zinc finger transcription factor that maintains the differentiated epithelial phenotype in renal cells (Jetten 2019). **C. CagA.** HPF57_0574. HP0547. The site 11-aa away from CBS is flanked by

conserved sites probably on a helix. **D. Zmp.** HPF57_1035. HP0382. It belongs to Peptidase family M48 (pfam), membrane zinc metallopeptidases with the characteristic HExxH motif. The *H. pylori* protein carries this motif (H271-H278 in HPF_1035) and also another E (E348) as another Zn coordinator from the next helix. The family include human ZMPSTE24/FACE-1 maturating lamin A for nuclear lamina and yeast Ste24p maturating a peptide pheromone. From CAAX (A = aliphataic) at C-terminus with isoprenoid attached on C, they cleave off AAX. Because cysteine isoprenylation has not been detected in prokaryotes, this enzyme may target human proteins such as lamin A. The site is on a cytoplasmic helix below a beta-sheet.

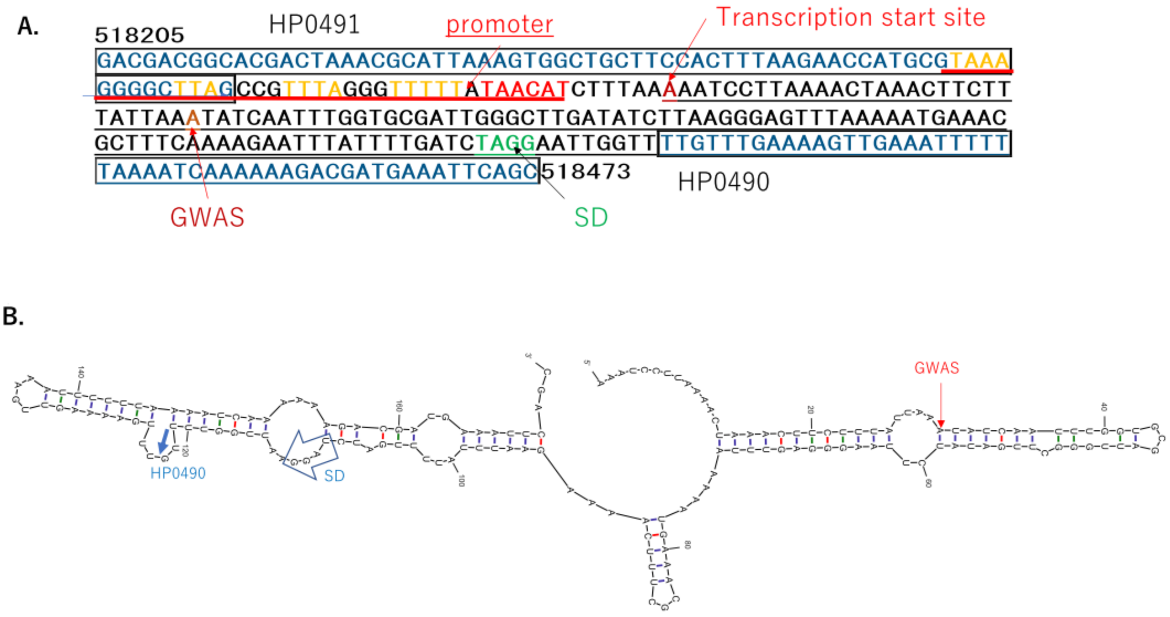

S10 Figure. GWAS SNP upstream of *kch*, *trkA* (HP0490). A. Sequence. **B.** Predicted secondary structure of the 5′ part of the expected mRNA.

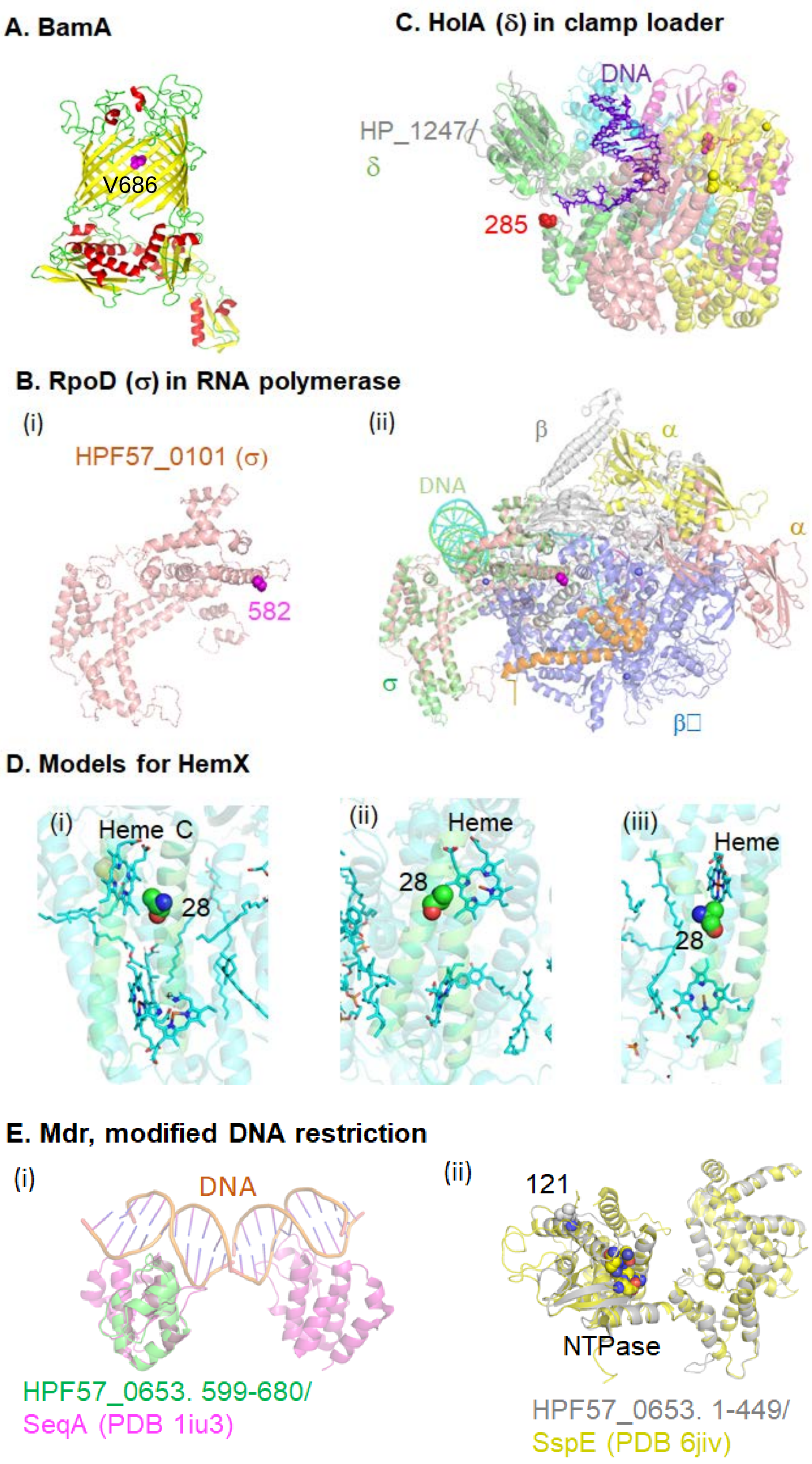

S11 Figure. Synonymous SNPs identified by GWAS. A. BamA (HPF57_0678) modeled on 5d0o. BamA forms part of the outer membrane protein assembly complex, which is involved

in assembly and insertion of beta-barrel proteins into the outer membrane in Gram-negative bacteria. BamA is also a part of contact-dependent growth inhibition (CDI) systems found among Gram-negative bacteria, which enables them to bind to neighboring bacterial cells and deliver protein toxins that inhibit cell growth. CDI-BamA receptor interaction provides a mechanism by which bacteria can distinguish siblings and other close relatives (self) from more distant relatives or other species of bacteria (nonself) (Ruhe, et al. 2013). C-half (493-) of this protein form a domain called Bacterial surface antigen D15 (IPR000184) or OMP85 (PF01103). This domain of several bacteria elicits immunity against them. V686 in magenta spheres is in the wall of the beta-barrel of this domain. **B.** RpoD (HPF57_0101) modeled on 4ylp (*E. coli* RNA polymerase). RpoD or σ70, a sigma factor, is involved in initiation of transcription through promoter selection in the normal exponential growth. S582 is in σD3 domain, which recognizes the extended −10 element (TG at positions −14 and −13) in *E. coli* while σD2 recognizes the −10 element of promoter and σD4 recognizes its −35 element (Chen, et al. 2021). S582 is at the C-terminus of σD3 and interacts with beta-beta′ subunit. **C.** HolA (HPF57_1206) in the clamp loader complex. HolA is a homolog of delta subunit of the clamp loader complex for DNA polymerase III of *Escherichia coli*. HP1247 was modeled on 1xxi and aligned with *E. coli* clamp loader (PDB 3gli). In the model, P285 is on its third domain interacting with the template DNA. **D.** HemX (HPF57_1042). SwissModel revealed structural similarity of HPF57_1042 to a trans-membrane helix pair in three respiratory-chain proteins (PDB 1q90, 6hwh, 1kb9). A28 in the three models appear to interact with a protoporphyrin ring. We expect that this protein interacts with a protoporphyrin ring. Consistent with this, upstream of HPF57_1042 lies ferrochelatase gene (*hemH*). We tentatively named this gene *hemX*. **E.** Mdr, modified-DNA specific restriction enzyme (HPF57_0653). HPF57_0653 has some structural similarity with SspE that restricts DNA with phosphorothioate modification (PDB 6jiv) (Xiong, et al. 2020) (ii), GmrSD that restricts

DNA with glucosylated hydroxymethylcytosine (HMC) (InterPro) and other members of His-Me finger endonuclease superfamily. Its C-terminus resembles SeqA that recognizes the hemimethylated G-mA-T-C sites in the *oriC* region of the *E. coli* chromosome (PDB 1iu3) (i). We expect that it restricts a modified DNA through strand cleavage and tentatively designate its gene as *mdr* for modified DNA restriction. The residue 121 and NTPase active-site amino-acids D96GQQR (both in spheres) are close.

Supplementary References

Bellesis AG, Jecrois AM, Hayes JA, Schiffer CA, Royer WE, Jr. 2018. Assembly of human C-terminal binding protein (CtBP) into tetramers. J Biol Chem 293:9101-9112.

Chen J, Boyaci H, Campbell EA. 2021. Diverse and unified mechanisms of transcription initiation in bacteria. Nat Rev Microbiol 19:95-109.

Chinnadurai G. 2009. The transcriptional corepressor CtBP: a foe of multiple tumor suppressors. Cancer Res 69:731-734.

Jetten AM. 2019. Emerging Roles of GLI-Similar Kruppel-like Zinc Finger Transcription Factors in Leukemia and Other Cancers. Trends Cancer 5:547-557.

King CR, Zhang A, Tessier TM, Gameiro SF, Mymryk JS. 2018. Hacking the Cell: Network Intrusion and Exploitation by Adenovirus E1A. mBio 9.

Nishimori I, Minakuchi T, Kohsaki T, Onishi S, Takeuchi H, Vullo D, Scozzafava A, Supuran CT. 2007. Carbonic anhydrase inhibitors: the beta-carbonic anhydrase from Helicobacter pylori is a new target for sulfonamide and sulfamate inhibitors. Bioorg Med Chem Lett 17:3585-3594.

Reusch RN. 2012. Insights into the structure and assembly of Escherichia coli outer membrane protein A. FEBS J 279:894-909.

Ruhe ZC, Wallace AB, Low DA, Hayes CS. 2013. Receptor polymorphism restricts contact-dependent growth inhibition to members of the same species. mBio 4.

Supuran CT, Capasso C. 2016. New light on bacterial carbonic anhydrases phylogeny based on the analysis of signal peptide sequences. J Enzyme Inhib Med Chem 31:1254-1260.

Xiong X, Wu G, Wei Y, Liu L, Zhang Y, Su R, Jiang X, Li M, Gao H, Tian X, et al. 2020. SspABCD-SspE is a phosphorothioation-sensing bacterial defence system with broad anti-phage activities. Nat Microbiol 5:917-928.
